# Single-Cell Transcriptome Profiles of Foxl1-Lineage Cells Along the Intestinal Crypt–Villus Axis

**DOI:** 10.1016/j.jcmgh.2026.101849

**Published:** 2026-07-16

**Authors:** Amal Gharbi, Noa Corem, Sharona Elgavish, Inbar Plachkes, Abrar Jamous, Marco Canella, Moriya Shmuel, Hadar Horowitz, Jianmei Tan, Hongqian Chen, Bing Su, Mahmud Abu-Gazala, Noam Shussman, Michal Shoshkes-Carmel

**Affiliations:** 1 Department of Developmental Biology and Cancer Research, Institute for Medical Research Israel-Canada, Hebrew University Medical School, Jerusalem, Israel; 2 Info-CORE, Bioinformatics Unit of the I-CORE at the Hebrew University of Jerusalem, Jerusalem, Israel; 3 Shanghai Institute of Immunology, Department of Immunology and Microbiology, Shanghai Jiao Tong University School of Medicine, Shanghai, China; 4 Department of General Surgery, Hadassah Medical Centre and Faculty of Medicine, Hebrew University of Jerusalem, Jerusalem, Israel

**Keywords:** Foxl1, Functional Zonation, Mesenchyme, Signaling Gradients, Stem Cells, Stem Cell Niche

## Abstract

**Background & Aims:**

Intestinal epithelial cells rely on a complex array of stromal signals to determine their fate and function along the crypt–villus axis, but the precise cellular sources and combinations of signals at each position remain poorly defined.

**Methods:**

We generated an atlas of Foxl1-lineage cells along the crypt–villus axis using single-cell RNA sequencing integrated with in situ hybridization, immunofluorescence, and reporter mouse models.

**Results:**

We identify 4 spatially distinct Foxl1-lineage subepithelial populations: crypt, villus–base, villus–mid and villus–tip, that segregate into 2 related pairs. Crypt and villus–mid Foxl1-lineage cells share a low platelet-derived growth factor receptor α transcriptional program enriched for canonical Wnts and R-spondins, consistent with regenerative signaling functions. In contrast, villus–base and villus–tip Foxl1-lineage cells are platelet-derived growth factor receptor α–high and express noncanonical Wnt5a and bone morphogenetic protein ligands. Crypt Foxl1-lineage cells are additionally enriched for extracellular matrix proteins and complement components, whereas villus–base Foxl1-lineage cells express contractility-associated genes, suggesting a role in villus architecture. Villus–mid Foxl1-lineage cells display signatures of immune regulation and inflammation, whereas villus–tip Foxl1-lineage cells are specialized for regulation of ribonucleoprotein complexes and nutrient sensing.

**Conclusions:**

This atlas provides a foundational resource for understanding how diverse stromal populations coordinate signaling niches and influence intestinal epithelial homeostasis.

## Graphical abstract

**Figure:**
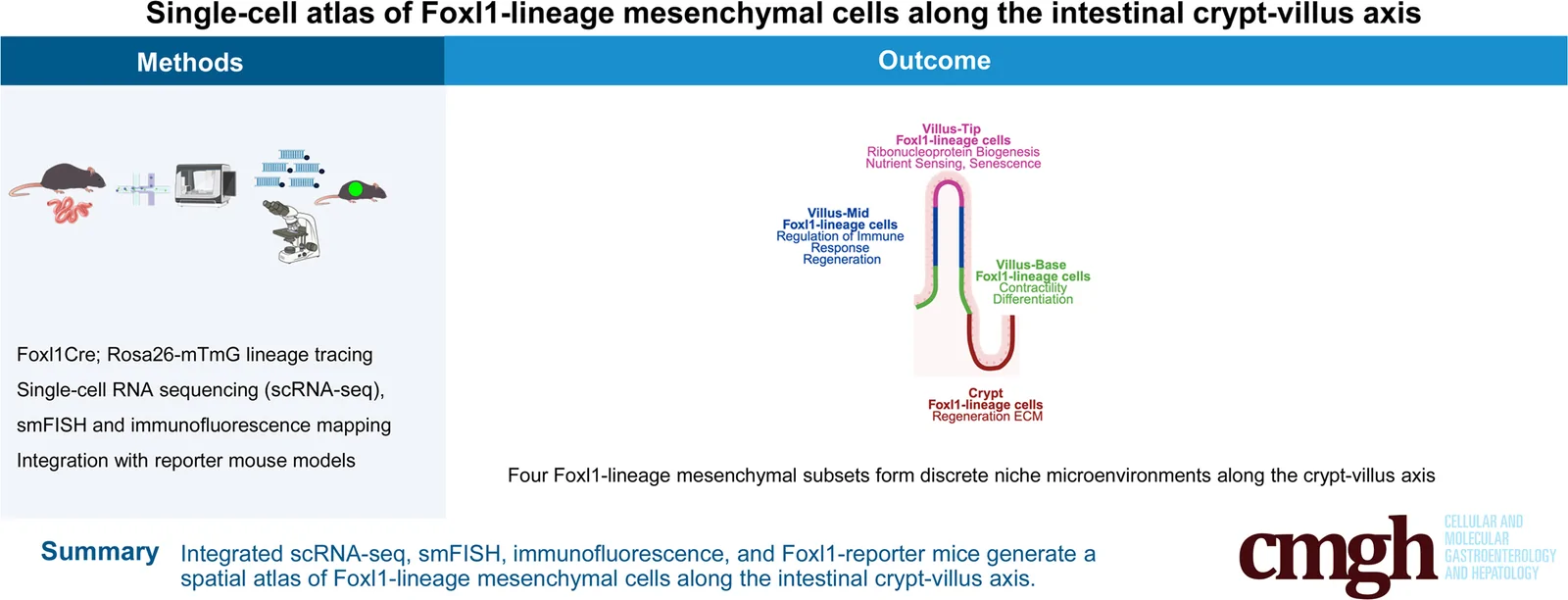

**Summary**

Foxl1-lineage cells form a subepithelial network that supports intestinal stem cells. Using single-cell RNA sequencing and in situ mapping, we identify 4 transcriptionally and spatially distinct Foxl1-lineage subtypes with specialized signaling, matrix, and immune programs that together shape regional epithelial niches.

**What You Need to Know**

**Background**

Foxl1-lineage cells form a subepithelial network along the intestinal crypt–villus axis that is essential for stem cell function. However, the diversity of these cells and their potential region-specific roles along the crypt–villus axis have remained largely unexplored.

**Impact**

We identify 4 Foxl1-lineage subtypes, each with a distinct gene signature and signaling profile. These findings point to new candidate mechanisms for intestinal tissue maintenance, immune regulation, and local epithelial–mesenchymal signaling.

**Future Directions**

Functional studies, including subtype-specific ablation, targeted genetic manipulations, and live imaging, will determine how individual Foxl1-lineage subtypes contribute to intestinal regeneration, stem cell support, epithelial renewal, immune signaling, and tissue repair.

The small intestine is lined by a single layer of epithelial cells organized into repeating crypt–villus units. This structured arrangement is crucial for protecting stem and progenitor cells in the crypt from the harsh conditions of the intestinal lumen, maximizing nutrient absorption, and maintaining a robust, continuous barrier against physical stress and invading pathogens.

Along the crypt–villus axis, specific epithelial cell types and functions are restricted to defined locations. Remarkably, this intricate pattern is preserved despite the intestine’s rapid and continuous renewal. Stem and progenitor cells at the crypt divide continuously to produce new cells, which then migrate upward along the villus and are eventually shed at the tip. As a result, the epithelial layer is in perpetual motion, yet the overall spatial organization remains stable. This stability is achieved because stromal signals along the crypt–villus axis repeatedly instruct epithelial cells to adopt specific roles as they migrate toward the villus tip.

Among intestinal stromal cells, Foxl1-lineage cells have been used to mark a subepithelial telocyte population, a mesenchymal cell type notable for its exceptionally long cellular projections known as telopodes.^1^, ^2^, ^3^, ^4^, ^5^, ^6^ Intestinal telocytes form an interconnected subepithelial network implicated in maintaining the stem cell pool and directing enterocyte differentiation.^6^, ^7^, ^8^, ^9^, ^10^ Telocytes are recognized for compartmentalizing signaling molecules along the crypt–villus axis.^7^^,^^8^ However, their molecular diversity necessitates single-cell resolution for full characterization. Despite advances in characterizing stromal cells,^8^^,^^11^, ^12^, ^13^, ^14^, ^15^ the low expression of Foxl1 and the molecular heterogeneity of Foxl1-lineage cells have limited their comprehensive analysis using conventional single-cell approaches.

To overcome these challenges, we employed a Foxl1Cre-driven reporter mouse model combined with single-cell RNA sequencing (scRNA-seq) and single-molecule RNA fluorescence in situ hybridization (smFISH) to enrich, visualize, and molecularly profile Foxl1-lineage cells in situ. We identify distinct subepithelial Foxl1-lineage subtypes with defined expression patterns along the crypt–villus axis, providing a framework for how stromal cells may contribute to intestinal epithelial cell fate decisions.

## Results

### Foxl1-Lineage Cells Form a Conserved Subepithelial Network From Crypt Base to Villus Tip

**Figure 1:**
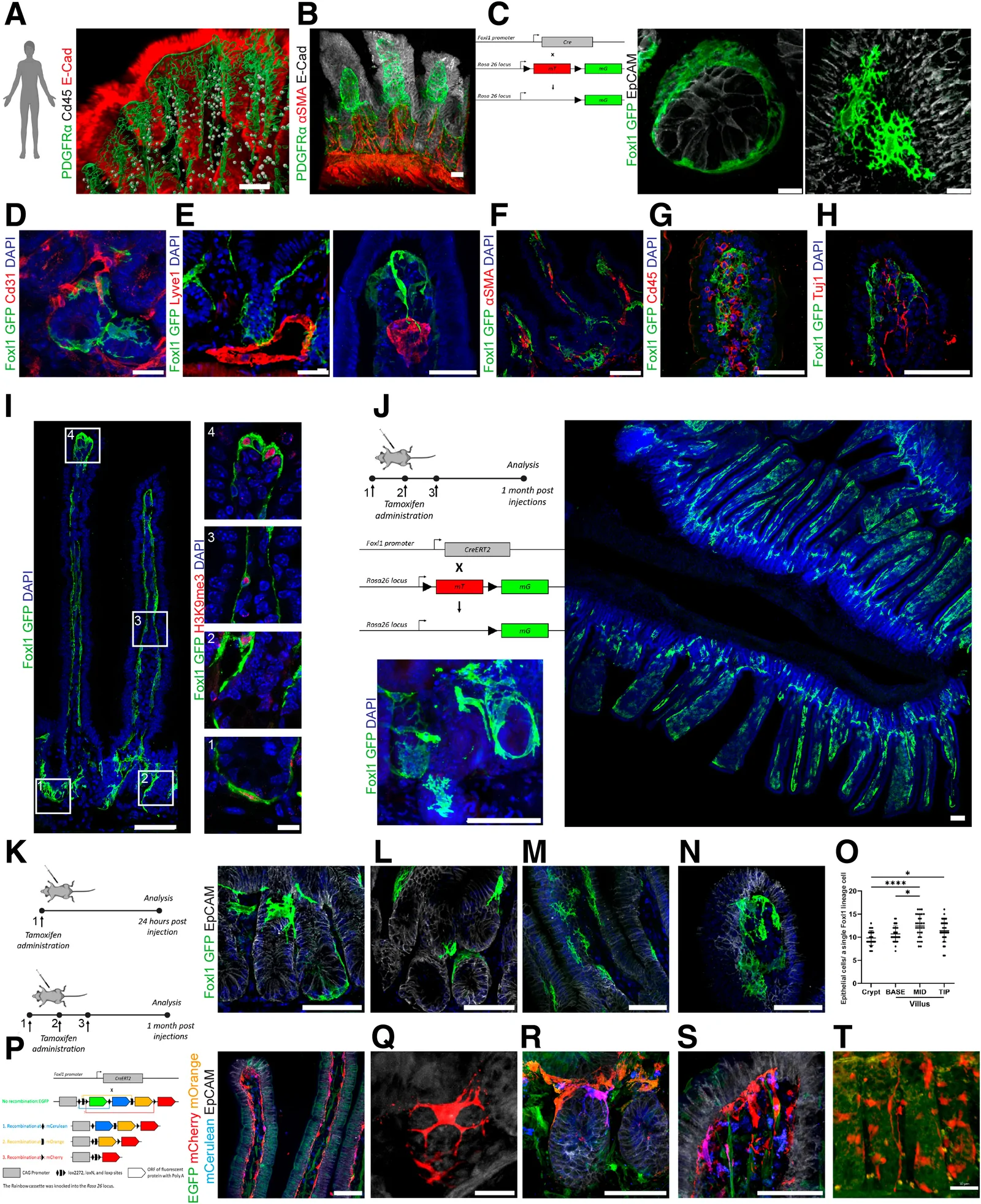
**Foxl1-lineage cells form a conserved subepithelial network from crypt base to villus tip.** (*A* and *B*) Whole-mount immunofluorescence of cleared human colon (*A*) and small intestine (*B*). Panel (*A*) shows a 3D volume reconstruction generated in IMARIS, and panel (*B*) shows a z-stack projection. PDGFRα expression highlights a continuous subepithelial network along colonic crypts and the crypt–villus axis of the small intestine. E-cadherin marks the epithelium, CD45 labels immune cells, and αSMA marks myofibroblasts, illustrating close spatial relationships between subepithelial PDGFRα+ cells, epithelial cells, immune cells, and myofibroblasts. (*C–I*) Schematic of membrane-bound GFP expression in Foxl1-lineage cells (Foxl1Cre; Rosa-mTmG). Immunofluorescence for GFP on 30-μm jejunum sections reveal subepithelial Foxl1-lineage cells as thin, flat cells in the crypt region (*C*, *left*) and thick, stellate cells in the villus region (*C*, *right*). High-resolution imaging shows GFP+ Foxl1-lineage cells in close proximity to blood vessels (CD31; *D*), lymphatic vessels (Lyve1; *E*, *left image crypt region, right image villus tip region*), myofibroblasts (αSMA; *F*), immune cells (CD45l; *G*), and neurons (Tuj1; *H*). Immunofluorescence for H3K9me3 demonstrates relatively high heterochromatin content in Foxl1-lineage nuclei along the crypt-villus axis (*I*). (*J*) Experimental design: tamoxifen induction in Foxl1CreERT2; Rosa-mTmG mice drives membrane-bound GFP expression in Foxl1-lineage cells. Immunofluorescence of 100-μm Foxl1CreERT2; Rosa-mTmG jejunal sections shows GFP+ Foxl1-lineage cells forming a subepithelial network along the crypt–villus axis. A zoomed view of the crypt region highlights the thin, flat morphology of Foxl1-lineage cells, similar to that observed in the Foxl1Cre; Rosa-mTmG model. (*K–N*) Schematic illustrating that a single tamoxifen induction labels Foxl1-lineage cells inefficiently in Foxl1CreERT2; Rosa-mTmG mice. Immunofluorescence for GFP reveals individually labeled Foxl1-lineage cells at the crypt–villus axis. (*O*) Quantification of epithelial cell contacts made by single Foxl1-lineage cells at defined positions along the crypt–villus axis, measured in 30-μm sections under inefficient Foxl1CreERT2; Rosa-mTmG and efficient Foxl1CreERT2; Rosa-Rainbow induction (n = 3 mice per group, <10 axes per bar; ∗∗∗∗*P* < .0001, unpaired 2-tailed *t* test). (*P–T*) Schematic of tamoxifen-induced expression of membrane-bound multispectral Rainbow fluorescence in Foxl1-lineage cells (Foxl1CreERT2; Rosa-Rainbow mice). Immunofluorescence of jejunal sections stained for red fluorescent protein (RFP) visualizes single Foxl1-lineage cells along the villus (*P*), crypt (*Q* and *R*), and villus tip (*S*). Whole-mount immunofluorescence of Foxl1CreERT2; Rosa-Rainbow colon stained for RFP highlights single Foxl1-lineage cells along colonic crypts (*T*). Scale bar, 50 μm. DAPI, 4′,6-diamidino-2-phenylindole.

Previous work showed that platelet-derived growth factor receptor α (PDGFRα)+ Foxl1-lineage cells form a continuous subepithelial network in mouse intestine,^7^^,^^8^ but it was unclear whether this organization is conserved in humans. To address this, we analyzed fresh tissue from normal margines of colectomies and intestinal resections, clearing both large and small intestine and staining for PDGFRα (Figure 1*A* and *B*). In the colon, PDGFRα+ cells formed a continuous network closely apposed to crypts (Figure 1*A*), whereas in the small intestine, the network extended along the entire crypt–villus axis (Figure 1*B*). This pattern mirrored that in mice, suggesting a conserved organization across species. In addition to their intimate association with the epithelium, PDGFRα+ cells contacted CD45+ immune cells (Figure 1*A*) and α smooth muscle actin (αSMA)+ myofibroblasts in the crypt region, whereas villus myofibroblasts localized to the villus core (Figure 1*B*).

To characterize the subepithelial network of Foxl1-lineage cells in mice in more detail, we used the Foxl1Cre line^16^ crossed to the Rosa-mTmG reporter.^17^ Foxl1-lineage cells associated with the crypt appeared flat and thin with extremely slender, short processes, whereas Foxl1-lineage cells along the villus exhibited a thick stellate morphology with multiple branched extensions (Figure 1*C* and Supplementary Videos 1–3), consistent with prior descriptions of a “pericryptal mesenchymal syncytium.”^18^ Foxl1-lineage cells maintained close associations with CD31+ blood vessels, Lyve1+ lymphatic vessels, αSMA+ myofibroblasts, CD45+ immune cells, and Tuj1+ neurons (Figure 1*D–H*), underscoring their potential role in coordinating epithelial, vascular, stromal, immune, and neuronal compartments. In line with electron microscopy studies on telocytes, Foxl1-lineage cell nuclei showed relatively high heterochromatin content, as indicated by H3K9me3 staining (Figure 1*I*).

We next analyzed an independent inducible Foxl1CreERT2; Rosa-mTmG mouse model.^19^ Given that mouse jejunal epithelial crypts are approximately 70 to 90 μm deep,^20^ we imaged 100-μm longitudinal sections. Three tamoxifen injections yielded robust membrane-tagged green fluorescent protein (GFP) labeling across the subepithelial network (Figure 1*J*), in contrast to the sparse recombination reported for Foxl1CreERT2; Rosa-YFP.^21^ This highlights the value of membrane-tagged reporters and thick sections for efficient visualization of Foxl1-lineage cells. To resolve individual cell architecture, we either applied a single low-dose tamoxifen induction (Figure 1*K–N*; Supplementary Videos 1 and 2) or used Foxl1CreERT2; Rosa-Rainbow^22^ mice with efficient induction to obtain multispectral labeling (Figure 1*P–T*; Supplementary Video 3). Individual Foxl1-lineage cells formed a thin layer at the epithelial base along the crypt–villus axis and adopted an orthogonal orientation at the villus tip (Figure 1*N* and *S*). Each Foxl1-lineage cell contacted on average ∼10 epithelial cells in the jejunum (Figure 1*O*; Methods), although precise counts are limited by their extremely fine, oriented processes. These features suggest that Foxl1-lineage cells may partition the crypt–villus axis into epithelial segments that help position local signaling domains; testing this hypothesis will require future functional studies.

Similar Foxl1-lineage cell morphologies were observed in mouse colon (Figure 1*T*), where cells exhibited small, flat bodies or elongated processes extending up to one-third of the crypt length. Together, these structural analyses reveal an intricate, conserved subepithelial Foxl1-lineage cell network that is well-positioned to coordinate complex signaling along the intestine.

### Single-Cell Transcriptomics Distinguishes Four Subepithelial Foxl1-Lineage Subtypes

**Figure 2:**
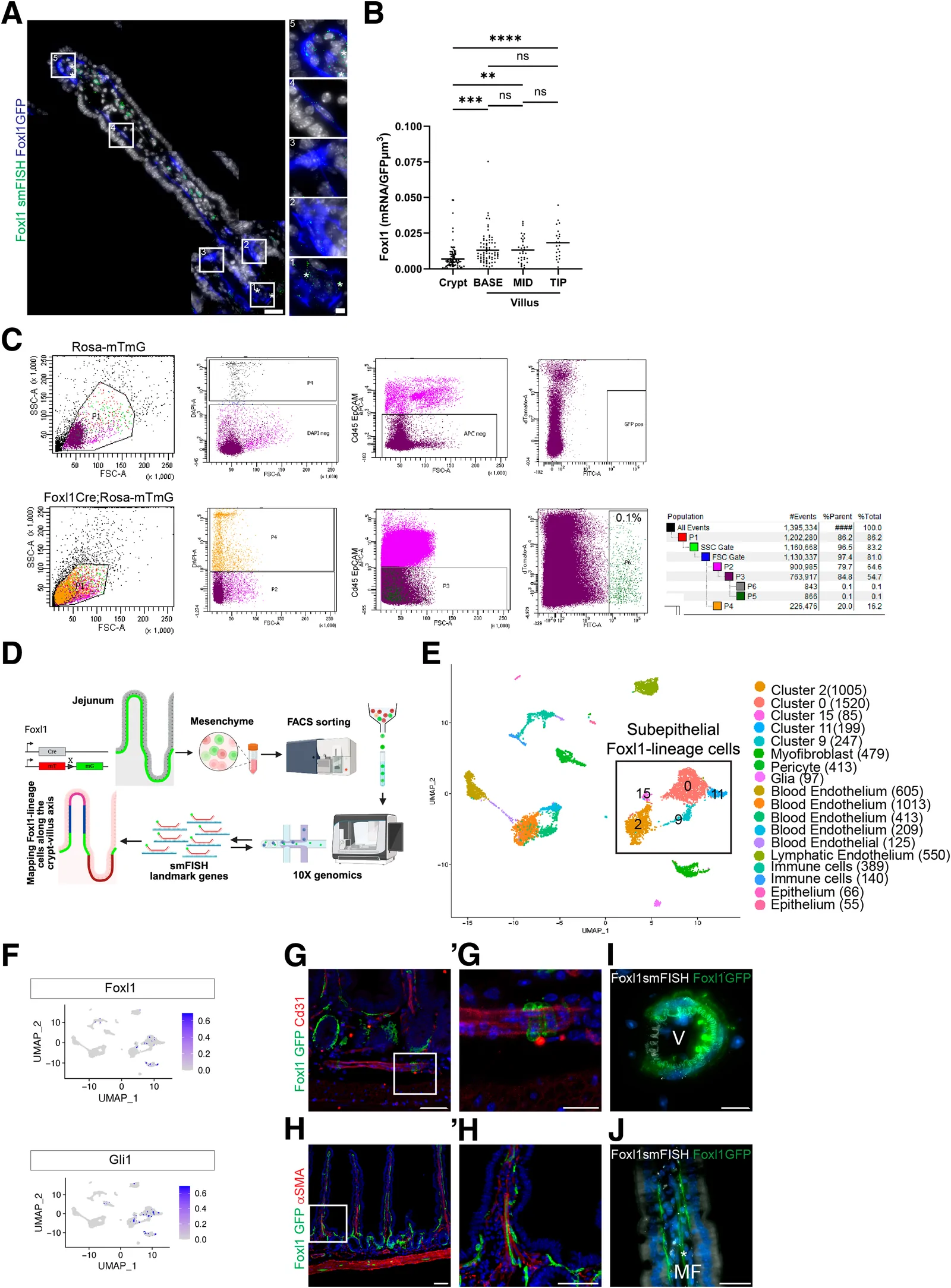
**Single-cell transcriptomics distinguishes 4subepithelial Foxl1-lineage subtypes.** (*A*) smFISH imaging of mouse jejunum sections from Foxl1Cre; Rosa-mTmG mice, hybridized for Foxl1 (*green dots*), demonstrates Foxl1 messenger RNA (mRNA) expression in GFP+ Foxl1-lineage cells (*blue*) positioned at the crypt, villus–base, villus–mid, and villus–tip. Scale bar, 10μm. (*B*) Quantification of Foxl1 mRNA concentrations in Foxl1-lineage cells along the crypt-villus axis by smFISH, revealing spatial variation (n = 3 mice per group; <10 crypt–villus axes per bar; ∗∗∗∗*P* < .0001; unpaired 2-tailed *t* test). (*C*) FACS plots from a representative Foxl1Cre; Rosa-mTmG mouse (7 mice analyzed) illustrating the gating strategy used to isolate GFP+ Foxl1-lineage cells. Jejunal tissue from Rosa-mTmG (GFP− control) and Foxl1Cre; Rosa-mTmG (GFP+) mice was dissociated, and 4′,6-diamidino-2-phenylindole (DAPI)-live singlets were gated. CD45+ immune cells and EpCAM+ epithelial cells were excluded, and in Foxl1Cre; Rosa-mTmG samples approximately 0.1% of total cells were GFP+ and sorted for downstream analysis. (*D*) Schematic illustrating the strategy used to generate a topologic map integrating single Foxl1-lineage cell transcriptional profiles with spatial position along the crypt−villus axis. Foxl1Cre; Rosa-mTmG mice were used for tissue dissociation, FACS isolation of GFP+ Foxl1-lineage cells, and smFISH mapping of cluster landmark genes. (*E*) UMAP projection of scRNA-seq data generated from GFP+ sorted jejunal cells (Foxl1Cre; Rosa-mTmG mice). A total of 7610 cells pooled from 7 mice were categorized into 18 clusters. (*F*) UMAP plots depict Foxl1 and Gli1 expression profiles across the 18 distinct clusters. (*G* and *H*) Immunofluorescence in Foxl1Cre; Rosa-mTmG mouse jejunum sections stained for Cd31 (*G*) and αSMA (*H*) reveals sparse GFP labeling in pericytes and myofibroblasts (MFs) alongside subepithelial Foxl1-lineage cells. (*I* and *J*) smFISH images of Foxl1Cre; Rosa-mTmG mouse jejunum, hybridized for Foxl1 (*white dots*), show Foxl1 mRNA in GFP+ (*green*) pericytes (*I*) surrounding large vessels (Vs) beneath the muscularis mucosa, and in MFs within the villus core (*J*; *asterisk* marks GFP-labeled MFs. Scale bar, 50 μm.

To assess whether Foxl1, a transcription factor important for Foxl1-lineage cell function and a marker for subepithelial telocytes,^23^, ^24^, ^25^, ^26^ is uniformly expressed along the crypt–villus axis, we used a dual approach: Foxl1Cre^16^; Rosa-mTmG reporter mice and smFISH to quantify Foxl1 transcripts (Figure 2*A*). This analysis revealed that villus Foxl1-lineage cells have higher Foxl1 transcript abundance than those in the crypt (Figure 2*B*).

To explore Foxl1-lineage cell heterogeneity, we performed scRNA-seq on jejunal tissue, leveraging the region’s well-characterized epithelial landscape along the villus axis.^27^ Mesenchymal cells from the jejunum of 7 mice were dissociated into single-cell suspensions, and Foxl1-lineage cells were enriched by fluorescence-activated cell sorting (FACS) GFP+ cells from Foxl1Cre; Rosa-mTmG mice, using Rosa-mTmG^17^ littermates (GFP−) as controls (Figure 2*C*). GFP+ cells from all 7 mice were sequenced using the 10X Genomics platform (Figure 2*D*), and the resulting datasets were integrated for joint analysis.

This strategy enriched Foxl1-lineage cells but did not yield a perfectly exclusive mesenchymal population. Prior PDGFRα+ sorting experiments, in which >80% of sequenced cells were epithelial or immune and only 329 bona fide stromal cells were recovered,^8^ illustrate the difficulty of isolating intestinal mesenchyme with high purity. In the present study, we therefore excluded epithelial cell adhesion molecules (EpCAM)+ and Cd45+ epithelial and immune cells from the GFP+ gate, generating a dataset largely depleted of these lineages. Endothelial contamination remained and could be further reduced by Cd31 depletion as described by Bernier-Latmani.^28^

In total, this workflow yielded approximately 4000 mesenchymal cells for downstream analysis (Figure 2*E*). Within the jejunal mesenchyme, we identified 3 major cell types: myofibroblasts, pericytes, and subepithelial Foxl1-lineage cells. Foxl1 and Gli1 transcripts, although generally low as expected for transcription factors, were enriched in mesenchymal populations relative to other lineages (Figure 2*F*), underscoring the value of mouse reporter models for reliable visualization of Foxl1-lineage cells. Sparse GFP labeling was also detected in pericytes associated with large vessels beneath the muscularis mucosae (Figure 2*G*) and in a subset of villus–base and mid–villus myofibroblasts (Figure 2*H*), both of which expressed Foxl1 (Figure 2*I* and *J*).

Unsupervised clustering revealed 5 distinct subepithelial clusters (Figure 2*E*). One cluster (cluster 9) showed low ribosomal protein gene expression and a reduced fraction of ribosomal gene-positive cells, suggesting compromised cell viability^29^ (Supplementary Table 1); this group was excluded from subsequent analyses.

### Single-Cell Transcriptomics Reveal Molecular Features of Crypt-Associated Foxl1-Lineage Cells in the Intestinal Stem Cell Niche

**Figure 3:**
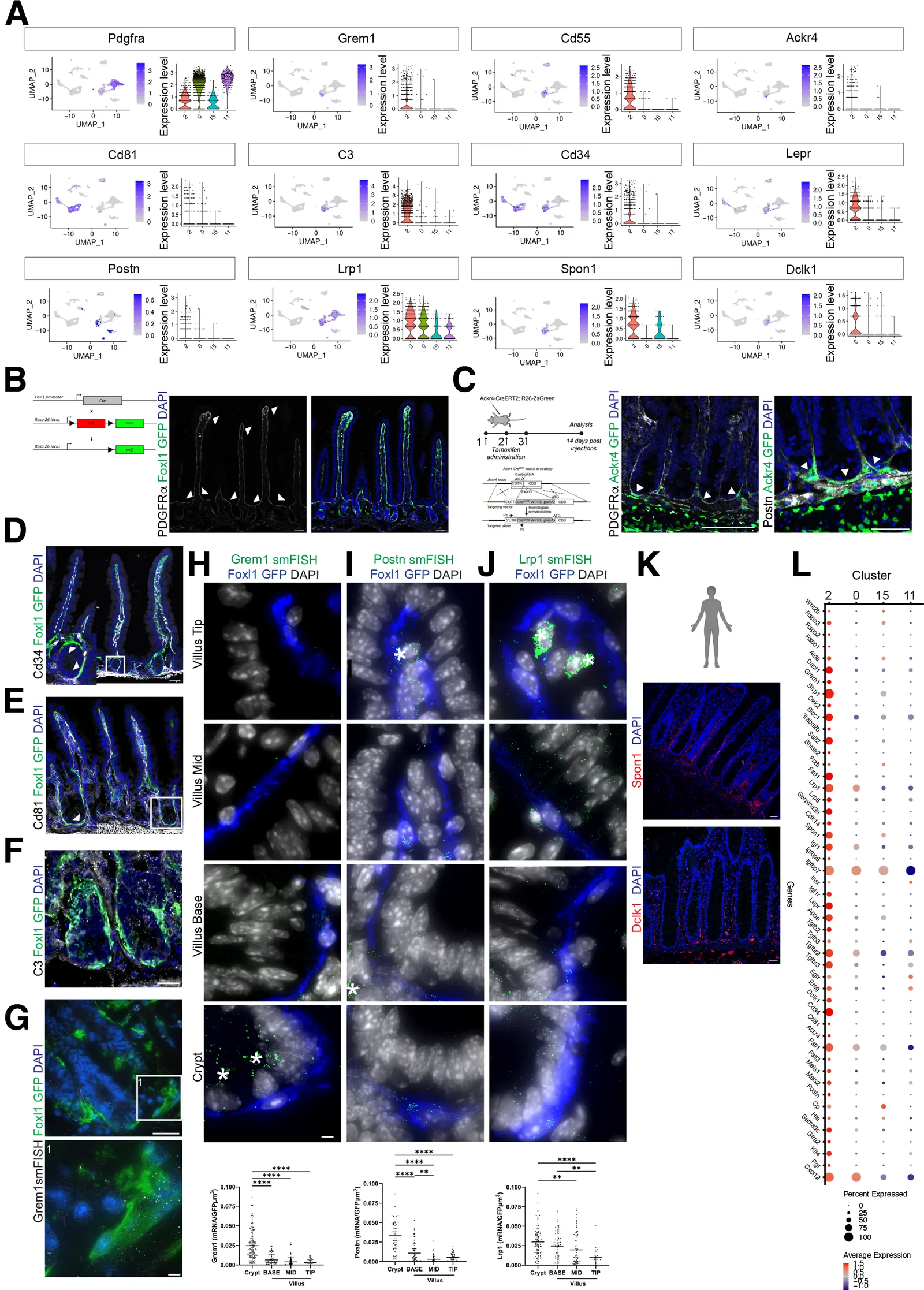
**Single-cell transcriptomics reveals molecular features of crypt-associated Foxl1-lineage cells in the intestinal stem cell niche.** (*A*) UMAP and violin plots display the expression patterns of landmark genes within the 18 clusters identified by scRNA-seq. (*B*) Immunofluorescence imaging of Foxl1Cre; Rosa-mTmG mouse jejunum sections stained for PDGFRα reveals 2 subepithelial Foxl1-lineage subpopulations: high PDGFRα expression at the villus tip and base, and low expression at the villus–mid region and crypt. (*C*) Schematic representation of the Ackr4CreERT2; Rosa26-ZsGreen mouse model, generated by CRISPR/Cas9 insertion of CreERT2 upstream of the Ackr4 ATG start codon. Immunofluorescence of jejunum sections from this reporter system, stained for GFP and PDGFRα or Postn, demonstrates localization of GFP+ trophocytes below the muscularis mucosa and GFP+PDGFRα+/Postn+ subepithelial cells at the crypt region (*arrowheads*). (*D–F*) Immunofluorescence analysis in Foxl1Cre; Rosa-mTmG mice stained for GFP and either CD34, Cd81, or C3 shows specific expression within crypt-associated Foxl1-lineage cells. (*G*) smFISH imaging of Foxl1Cre; Rosa-mTmG mouse jejunum hybridized for Grem1 highlights enriched Grem1 transcript patterns in GFP+ crypt-associated Foxl1-lineage cells. (*H–J*) smFISH images and quantification (n = 3 mice/group; <10 crypt–villus axes/bar; ∗∗∗∗*P* < .0001; unpaired 2-tailed *t* test) of Foxl1Cre; Rosa-mTmG mouse jejunum hybridized for Grem1, Postn, or Lrp1 (*green dots*), show specific enrichment of these transcripts in crypt-associated Foxl1-lineage cells (*blue*). *Asterisks* mark nonspecific autofluorescence from immune cells or Paneth cell granules. Scale bar, 10μm. (*K*) Immunofluorescence of human colon sections stained for Spon1 or Dclk1 demonstrates subepithelial crypt base localization. (*L*) Dot plot depicts stem cell niche factor expression in cluster 2 (crypt-associated Foxl1-lineage cells), with *dot size* denoting the percentage of Foxl1-lineage cells expressing each gene and *color* indicating expression levels within the cluster. Scale bar, 50μm; smFISH, 10μm. DAPI, 4′,6-diamidino-2-phenylindole.

Among the 4 subepithelial Foxl1-lineage clusters representing viable cells, PDGFRα expression segregated into 2 levels (Figure 3*A*): high in clusters 0 and 11, and low in clusters 2 and 15. Immunostaining of jejunal sections from Foxl1Cre; Rosa-mTmG mice confirmed this heterogeneity at the protein level (Figure 3*B*): subepithelial Foxl1-lineage cells at the villus tip and base expressed high PDGFRα, whereas Foxl1-lineage cells in the villus–mid and crypt regions showed lower levels.

On this basis, we divided the crypt–villus axis into 4 domains (crypt, villus–base, villus–mid, and villus–tip) and examined niche-associated markers. Cluster 2 was enriched for Grem1, Cd55, Ackr4, Cd81, and C3, genes previously linked to mesenchymal “trophocytes” beneath the muscularis mucosae,^11^^,^^12^ as well as Cd34 and Lepr, which have been associated with stem cell niche function^30^^,^^31^ (Figure 3*A*). These features initially raised the possibility that cluster 2 might include cells related to trophocytes.

To distinguish between these possibilities, we analyzed an Ackr4-CreERT2 reporter mouse. As reported, Ackr4+/GFP+ cells were abundant beneath the muscularis mucosae, consistent with trophocytes^11^^,^^12^ (Figure 3*C*). However, we also observed subepithelial GFP+ cells along the crypts that coexpressed PDGFRα and Postn (Figure 3*C*, *arrowhead*), consistent with crypt-associated Foxl1-lineage cells. Thus, markers such as Ackr4 can label both submuscularis trophocytes and crypt-associated Foxl1-lineage cells.

In Foxl1Cre; GFP-labeled jejunum, immunostaining for Cd34, Cd81, and C3 (Figure 3*D–F*), together with smFISH for Grem1, Postn and Lrp1 (Figure 3*G–J*), localized cluster 2 markers to Foxl1-lineage cells along the crypt epithelium. Grem1 transcripts were detected in GFP+ cells along the crypt, best appreciated along the subepithelial surface, with additional signal beneath the muscularis mucosa resembling the pattern described for trophocytes^11^ (Figure 3*G*). In human colon, staining for Spon1 and Dclk1, both enriched in cluster 2, showed signal at the crypt base (Figure 3*K*), supporting the assignment of cluster 2 as a crypt-associated Foxl1-lineage population.

Crypt-associated Foxl1-lineage cells were enriched for canonical stem cell niche factors, including Wnt and R-spondin pathway components, as well as IGF, EGF, TGFβ, and additional trophic factors (Figure 3*L*). They also expressed genes previously implicated in stem cell support and colorectal cancer stroma. Together, these transcriptomic and histologic data suggest how crypt-associated subepithelial Foxl1-lineage cells contribute to stem cell niche activity, in a manner consistent with prior mouse genetic studies in vivo.^7^^,^^9^^,^^11^^,^^30^, ^31^, ^32^

### Crypt-Associated Foxl1-Lineage Cell Transcriptomes Most Closely Resemble Stromal Cells Previously Identified as Trophocytes

**Figure 4:**
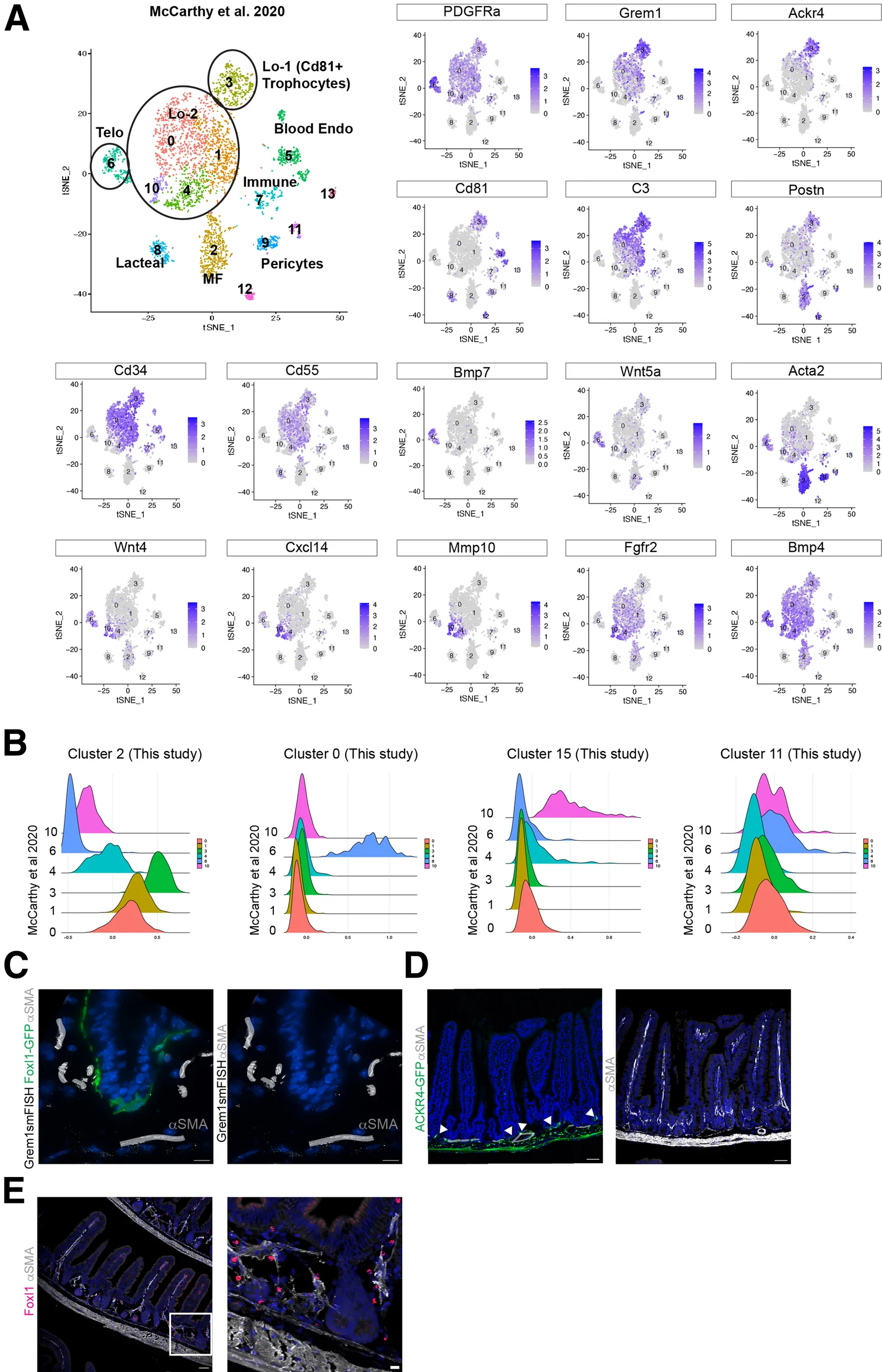
**Crypt-associated Foxl1-lineage cell transcriptomes most closely resemble stromal cells previously identified as trophocytes.** (*A*) t-Distributed stochastic neighbor embedding (tSNE) projection of scRNA-seq data from McCarthy et al (GEO: GSM4196131 and GSM4196132), processed using the published pipeline. Expression patterns of landmark genes are shown within the 14 clusters identified by scRNA-seq. (*B*) Ridge plot visualizations comparing 4 Foxl1-lineage clusters from this study with data from McCarthy et al indicate crypt-associated Foxl1-lineage cluster 2 align most closely with 3 PDGFRα low stromal clusters (clusters 3, 1, and 0) in the McCarthy dataset. Cluster 6 from the McCarthy dataset, previously annotated as telocytes, showed the strongest similarity with the Foxl1-lineage cluster 0 from this study, whereas cluster 15 matched most closely to McCarthy cluster 10 and also showed some similarity to cluster 4. (*C*) smFISH imaging of mouse jejunum sections from Foxl1Cre; Rosa-mTmG mice hybridized for Grem1 (*white dots*) and stained for αSMA (*gray outline*), illustrates how membrane GFP labeling enables visualization of Foxl1-lineage cells and assignment of transcripts to their cellular source. Grem1 mRNA is detected in GFP+ Foxl1-lineage cells at the crypt above the muscularis mucosa, as well as in cells located beneath the muscularis mucosa. (*D*) Immunofluorescence of mouse jejunum serial sections from Ackr4CreERT2; Rosa26-ZsGreen mice stained for αSMA (*gray outline*), shows GFP signal distribution primarily beneath the muscularis mucosa, whereas a small fraction is subepithelial above the muscularis mucosa that is consistent with Foxl1-lineage cells (*arrowhead*). (*E*) Immunofluorescence of mouse jejunum sections stained for Foxl1 (*red*) and αSMA (*white*) shows Foxl1+ nuclei (*red*) predominantly located above the muscularis mucosa. Scale bar, 50 μm.

To directly compare crypt-associated Foxl1-lineage cell transcriptomes with previously described trophocytes, we compared our single-cell dataset with the published trophocyte dataset (GEO: GSM4196131 and GSM4196132). Cells were filtered following McCarthy et al, retaining only those with <10% mitochondrial transcripts, >1500 detected genes, and genes expressed in at least 100 cells. After merging, scaling, and joint clustering using the top 50 principal components (resolution 0.4) and removing clusters expressing the hematopoietic marker Ptprc (CD45), we identified 14 clusters (Figure 4*A*). Six clusters from the McCarthy dataset showed high transcriptional similarity to our subepithelial Foxl1-lineage clusters, including the PDGFRα-low clusters Lo-1 and Lo-2 (clusters 3, 0, 1, 4,10) and the PDGFRα-high cluster annotated as telocytes (Figure 4*A*).

We then calculated module scores for each of our 4 Foxl1-lineage clusters using Seurat’s AddModuleScore function and visualized these scores as RidgePlot (Figure 4*B*). Expression of key Foxl1-lineage markers in cluster 2: Grem1, Ackr4, Cd81, C3, Postn, Cd34, and Cd55 (Figure 4*A*), and the corresponding module scores (Figure 4*B*) aligned most closely with 3 PDGFRα-low stromal clusters (clusters 3, 1, and 0), encompassing both Cd81+ trophocytes (cluster 3) and Cd81− stromal cells^12^ (clusters 1 and 0). The McCarthy cluster annotated as telocytes (cluster 6), showed the strongest similarity to our Foxl1-lineage cluster 0, whereas our cluster 15 most closely matched McCarthy cluster 10, with additional similarity to cluster 4. We did not identify a clear counterpart for our cell cluster 11, and no cluster expressing Ackr4 and Grem1 segregated cleanly from Foxl1-lineage cells.

To examine the histologic relationship between trophocytes and crypt-associated Foxl1-lineage cells, we combined these transcriptomic comparisons with reporter lines and in situ labeling. Using Foxl1Cre; Rosa-mTmG mice together with smFISH for Grem1, Ackr4CreERT2; R26-ZsGreen mice, and Foxl1 immunostaining, each colabeled with αSMA to mark the muscularis mucosa, we found that Grem1 transcripts and Ackr4-GFP signal were present both above and beneath the αSMA+ muscularis mucosa. In contrast, Foxl1+ nuclei were largely restricted to the subepithelial compartment above the muscularis mucosa (Figure 4*C–E*). These observations support the scRNA-seq finding that Grem1 and Ackr4 are expressed in crypt-associated Foxl1-lineage cells and indicate that Grem1/Ackr4–based reporter lines label both submuscularis trophocytes and crypt-associated Foxl1-lineage cells, whereas Foxl1 reporters predominantly label subepithelial cells above the muscularis mucosa.

Notably, Foxl1 transcripts were not detected in the filtered McCarthy dataset, consistent with the absence of a clearly resolved Foxl1+ telocyte population in that study and emphasizing the value of genetically engineered, membrane-tagged reporters for visualizing subepithelial Foxl1-lineage cells and assigning transcripts to specific stromal compartments.

### Crypt and Villus–Mid Foxl1-Lineage Cells, and Villus–Base and Villus–Tip Foxl1-Lineage Cells, Are Transcriptionally Paired Despite Spatial Separation

**Figure 5:**
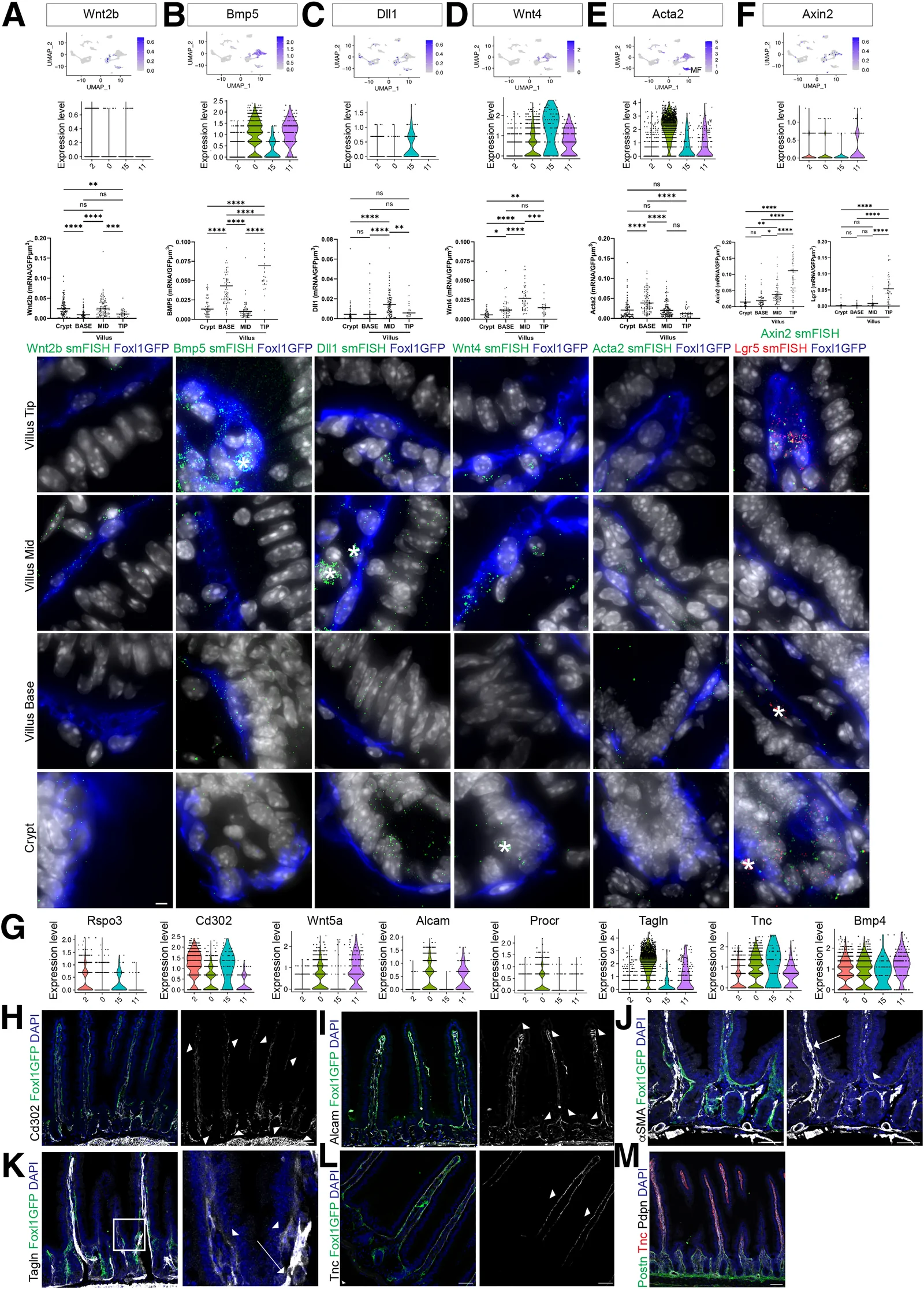
**Crypt and villus–mid Foxl1-lineage cells, and villus–base and villus–tip Foxl1-lineage cells, are transcriptionally paired despite spatial separation.** (*A–F*) UMAP projections, violin plots, and smFISH quantification/images reveal messenger RNA (mRNA) concentrations for landmark genes within GFP+ Foxl1-lineage cells (*blue*), located at the crypt, villus–base, villus–mid, and villus–tip. Genes visualized include Wnt2b (*A*), Bmp5 (*B*), Dll1 (*C*), Wnt4 (*D*), Acta2 (*E*), Axin2 (*green dots*), and Lgr5 (*red dots*) (*F*). Quantification shows significant variation between clusters along the axis (n = 3 mice/group; <10 axes/bar; ∗∗∗∗*P* < .0001; unpaired 2-tailed *t*-test). *Asterisks* denote non-specific autofluorescence from immune cells or Paneth cell granules. Scale bar, 10 μm. (*G*) Violin plots show landmark gene expression in Foxl1-lineage clusters, demonstrating complementary patterns: Wnt2b, Rspo3, and Cd302 are enriched in crypt and villus–mid Foxl1-lineage cells (clusters 2 and 15), whereas Bmp5, Wnt5a, and Alcam are enriched at the villus base and tip (clusters 0 and 11). (*H–M*) Immunofluorescence in mouse jejunum sections from Foxl1Cre; Rosa-mTmG (*H–L*) or C57Bl/6 (*M*) mice stained for marker combinations. Cd302 is enriched in Foxl1-lineage cells at the crypt and villus–mid regions (*H*), Alcam is enriched at villus–base and tip Foxl1-lineage cells (*I*), αSMA and Tagln are enriched at villus–base Foxl1-lineage cells (*J* and *K*), Tnc at villus–mid Foxl1-lineage cells (*L*), and Postn, Tnc, Pdpn at various regions (*M*). *Arrowheads* highlight expression in Foxl1-lineage cells; *arrows* indicate expression in myofibroblasts (MFs). Scale bar, 50μm; smFISH, 10μm. DAPI, 4′,6-diamidino-2-phenylindole.

Turning to villus Foxl1-lineage cells, we observed a distinct enrichment pattern localized to the villus–mid region, corresponding to cluster 15 (Figures 3*L* and 5*A*, *C*, *D*, and *G*). Cluster 15 showed strong transcriptional similarity to crypt-associated Foxl1-lineage cells (cluster 2). In contrast, clusters 0 and 11 displayed a complementary pattern, with elevated expression of Bmp5 and the noncanonical Wnt5a (Figure 5*B* and *G*). This opposition extended to PDGFRα, the adhesion molecule Alcam, Spon1, and the lectin receptor Cd302 (Figures 3*A* and 5G): PDGFRα and Alcam were highest in villus–base and villus–tip Foxl1-lineage cells (clusters 0 and 11) and lower in crypt and villus–mid Foxl1-lineage cells (clusters 2 and 15), whereas Cd302 and Spon1 showed the inverse distribution.

Immunostaining of jejunal sections from Foxl1Cre; Rosa-mTmG mice corroborated these patterns. Cd302 was strongly expressed in crypt and villus–mid Foxl1-lineage cells (Figure 5*H*), whereas Alcam was predominantly expressed in villus–base and villus–tip Foxl1-lineage cells, with lower levels in the crypt and villus–mid regions (Figure 5*I*). smFISH further confirmed complementary Wnt and bone morphogenetic protein (BMP) expression: Wnt2b was enriched in crypt and villus–mid Foxl1-lineage cells, whereas Bmp5 was enriched in villus–base and villus–tip Foxl1-lineage cells (Figure 5*A* and *B*). Bmp4 was detected across all subepithelial Foxl1-lineage populations, with the highest levels at the villus tip (Figure 5*G*). Together, these data indicate 2 transcriptionally related Foxl1-lineage groups: crypt/villus–mid and villus–base/villus–tip, that form opposing signaling domains along the crypt–villus axis.

Beyond Wnt2b and Rspo3, the villus-mid cluster (15) was also enriched for the Notch ligand Dll1, the Wnt ligand Wnt4, and the extracellular matrix protein tenascin-C (Tnc) (Figure 5*C*, *D*, and *G*). smFISH for Dll1 and Wnt4 localized these transcripts to the villus–mid region (Figure 5*C* and *D*), and immunostaining for Tnc confirmed the highest protein levels in the villus–mid (Figure 5*L*).

Although Acta2 (αSMA) and Tagln were most strongly expressed in myofibroblasts, both genes were also detected in subepithelial Foxl1-lineage cells (Figure 5*E*, *J*, and *K*). Expression was minimal in cluster 2 (crypt Foxl1-lineage cells) and highest in cluster 0 (villus–base Foxl1-lineage cells), albeit at levels well below those in myofibroblasts. smFISH for Acta2 (Figure 5*E*) and immunostaining for αSMA and Tagln (Figure 5*J* and *K*) support the assignment of cluster 0 to villus-base Foxl1-lineage cells. Cluster 0 was further marked by enrichment of the protein C receptor Procr (Figure 5*G*), whereas cluster 11 was characterized by high Axin2 expression (Figure 5*F*). smFISH for Axin2 and Lgr5 showed that villus–tip Foxl1-lineage cells exhibited the highest levels of both markers (Figure 5*F*).

Taken together, these analyses map cluster 2 to crypt Foxl1-lineage cells, cluster 0 to villus–base Foxl1-lineage cells, cluster 15 to villus–mid Foxl1-lineage cells, and cluster 11 to villus–tip Foxl1-lineage cells, and suggest that crypt/villus–mid and villus–base/villus–tip Foxl1-lineage cells form 2 transcriptionally related pairs that help establish regional signaling environments along the crypt–villus axis.

### Differential Gene Expression Analysis Suggests Distinct Functions of Foxl1-Lineage Cells Along the Crypt–Villus Axis

**Figure 6:**
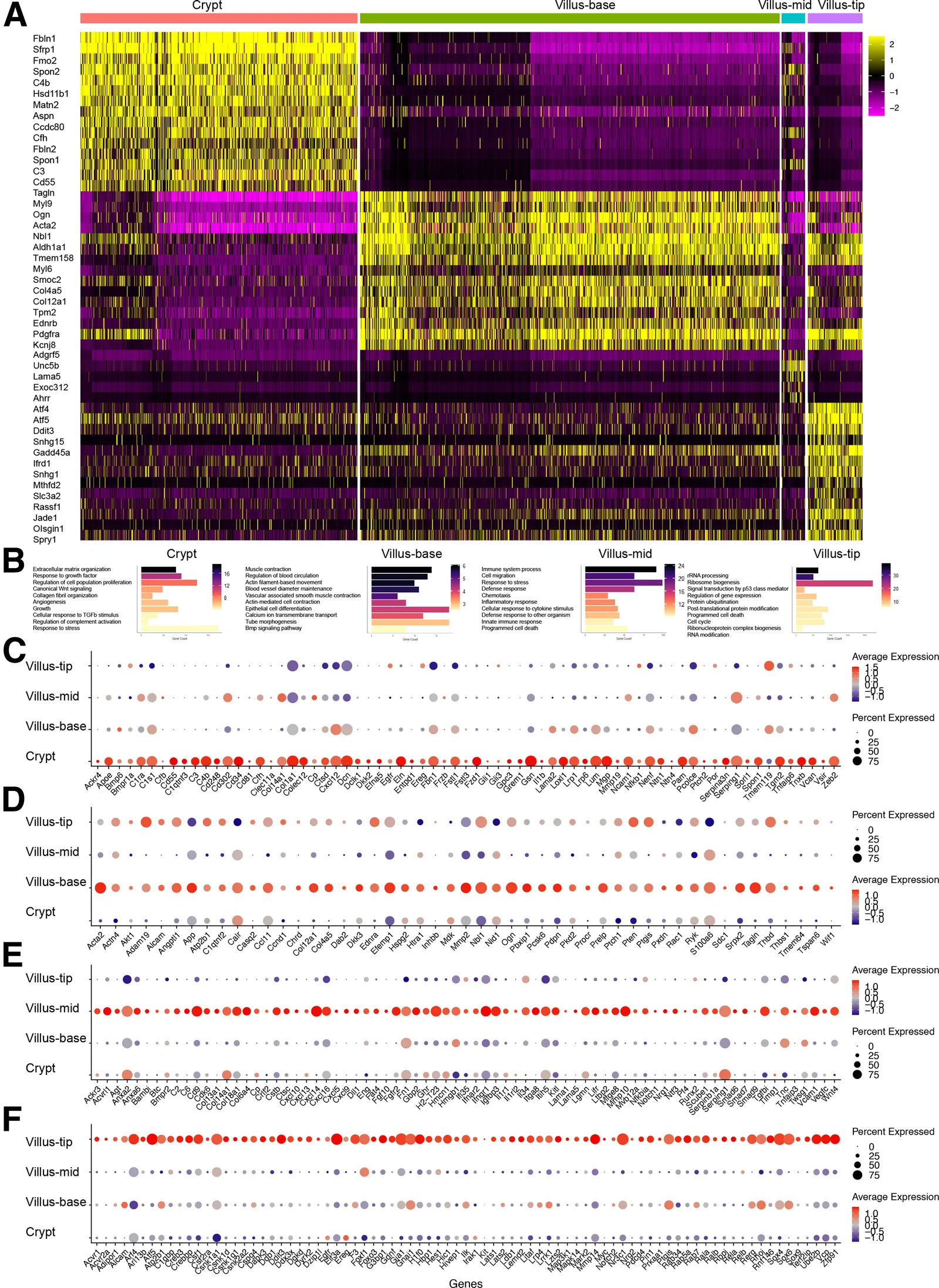
**Differential gene expression analysis suggests distinct functions of Foxl1-lineage cells along the crypt-villus axis.** (*A*) Heatmap showing the main differentially expressed genes in Foxl1-lineage subtypes located at the crypt, villus–base, villus–mid, and villus–tip. Each *column* represents a single cell, and each *row* represents a gene. (*B*) Gene Ontology pathway enrichment analyses of differentially expressed genes in Foxl1-lineage subtypes at the crypt, villus–base, villus–mid, and villus–tip (biological process). Scale bars are −Log_10_ (adjusted *P* value). (*C−F*) Dot plots showing the expression of unique genes in Foxl1-lineage cells located at the crypt, villus–base, villus–mid, and villus–tip. Each *dot* represents a gene, with *dot size* indicating the percentage of Foxl1-lineage cells expressing that gene, and *color* reflecting the expression level within each Foxl1-lineage subtype. This visualization highlights the spatial heterogeneity of gene expression among Foxl1-lineage populations along the crypt–villus axis.

Having identified and spatially mapped 4 Foxl1-lineage populations along the crypt–villus axis, we next performed differential gene expression analysis across these clusters (Figure 6*A–F*). Heatmap visualization of the most variable transcripts revealed clear subtype-specific patterns (Figure 6*A*). As expected, crypt and villus Foxl1-lineage cells differed markedly, and additional differences were evident among the villus subtypes.

Crypt-associated Foxl1-lineage cells expressed multiple components of canonical Wnt signaling together with inhibitors of Wnt and BMP pathways (including Sfrp1 and Grem1), Hedgehog-interacting protein, and elements of the transforming growth factor β (TGFβ) pathway (Figure 6*A–C*). These cells showed low PDGFRα expression, consistent with a specialized niche role. Crypt-associated Foxl1-lineage cells were also enriched for genes involved in extracellular matrix (ECM) remodeling,^33^ cell–substrate adhesion, and filament organization, as well as enzymes linked to stress resistance, xenobiotic detoxification, lipoprotein assembly (ApoE), and prostaglandin signaling (Ptger1, Ptger4, Ptgr1 and Ptges (Supplementary Table 2).

At the crypt–villus interface, villus–base Foxl1-lineage cells displayed a transcriptional program enriched for contractility-related genes, including Acta2 (αSMA), myosin light chain, tropomyosin, and endothelin receptors A and B^34^ (Figure 6*A*, *B*, and *D*), consistent with a structural contribution to crypt–villus architecture. These cells also expressed genes involved in actin cytoskeleton regulation, tube morphogenesis, and control of blood vessel diameter. Notably, villus–base Foxl1-lineage cells were enriched for genes in retinoic acid metabolism and BMP signaling (Aldh1a1, Aldh1a2, Aldh2, Bmp3, Bmp5, Bmp6, Bmp7), suggesting that they may contribute to the villus-to-crypt BMP gradient that shapes epithelial differentiation.

Villus–mid Foxl1-lineage cells, although small in number, showed a profile indicative of immune surveillance and inflammatory regulation, with increased expression of genes involved in chemokine and cytokine signaling, host defense, and both innate and adaptive immunity (Figure 6*A*, *B*, and *E*). Pathway analysis highlighted signatures related to interferon responses, interleukin6/Janus kinase/signal transducer and activator of transcription 3, and TGFβ signaling, consistent with a role in modulating inflammatory cues and tissue homeostasis.

Villus–tip Foxl1-lineage cells exhibited a distinct transcriptional landscape enriched for genes involved in ribonucleoprotein complexes, ribosome biogenesis, nutrient sensing, and autophagy (Figure 6*A*, *B*, and *F*). They also expressed high levels of transcription factors such as Foxf1 (closely associated with Foxl1),^35^^,^^36^ nuclear factor κB and p53, together with genes linked to the tumor necrosis factor α, mechanistic target of rapamycin, and Wnt/β-catenin signaling, suggesting functions in metabolic adaptation and stress responses. In addition, villus–tip Foxl1-lineage cells were enriched for components of chromatin-remodeling and polycomb complexes, as well as genes involved in protein ubiquitination and other post-translational modifications, pointing to potential roles in regulating gene and protein activity and supporting epithelial integrity.

### Spatially Zonated Potential Functions of Intestinal Foxl1-Lineage Cells: Growth, Matrix, and Immune Regulation Along the Crypt–Villus Axis

**Figure 7:**
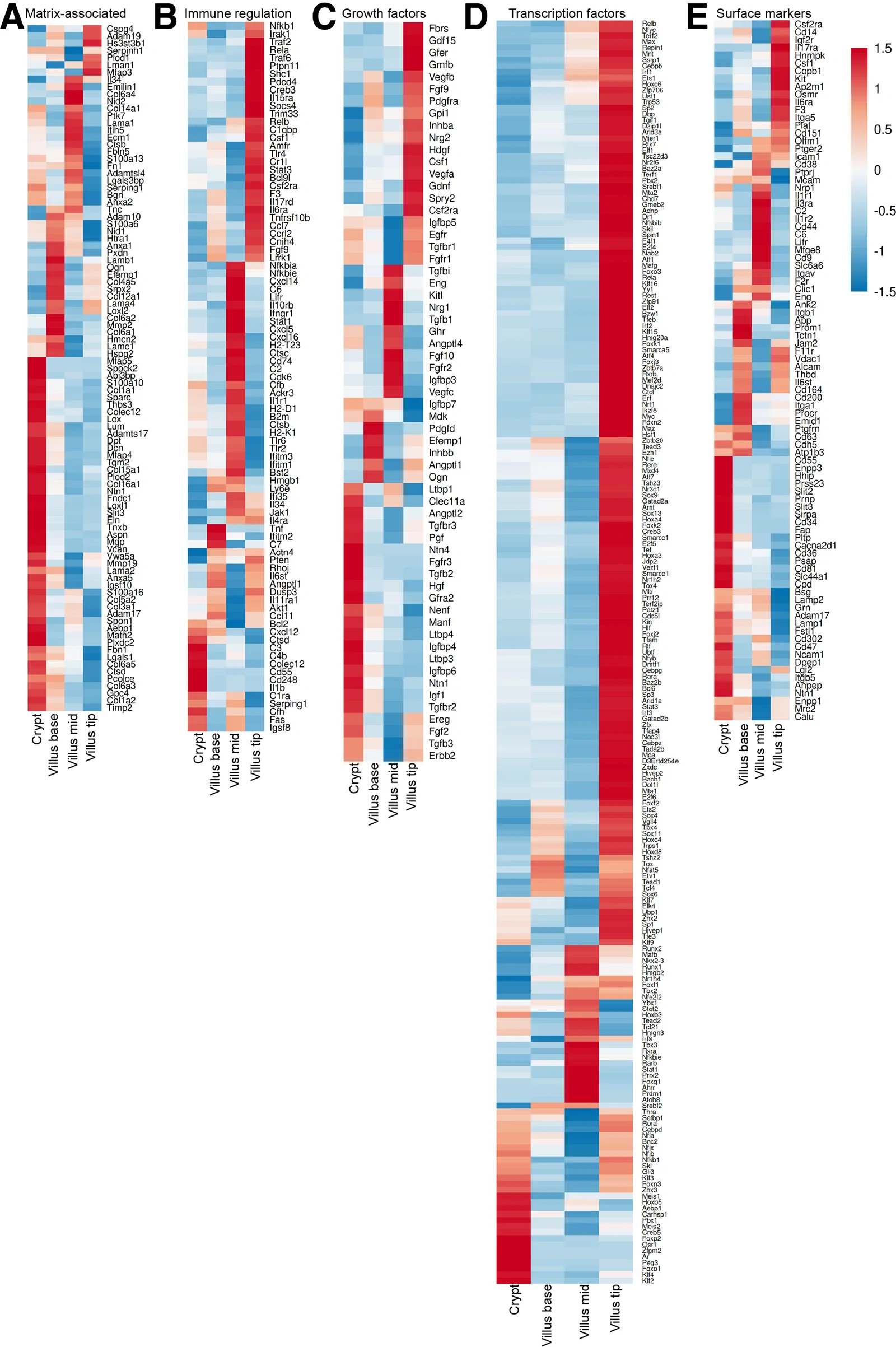
**Spatially zonated potential functions of intestinal Foxl1-lineage cells: growth, matrix, and immune regulation along the crypt-villus axis.** (*A–E*) Heatmaps of scRNA-seq data show expression patterns of matrix-associated genes (*A*), immune-regulatory genes (*B*), growth factors (*C*), transcription factors (*D*), and surface markers (*E*) in Foxl1-lineage subtypes located at the crypt, villus–base, villus–mid, and villus–tip.

Foxl1-lineage subtypes showed distinct ECM gene expression profiles (Figure 7*A*). Crypt-associated Foxl1-lineage cells were enriched for genes involved in collagen Ι synthesis and filamentous network assembly, whereas villus Foxl1-lineage cells preferentially expressed basement-membrane components. Immune-related genes also followed a zonated pattern (Figure 7*B*): villus–tip Foxl1-lineage cells were enriched for genes mediating pathogen recognition and innate immune activation, villus–mid Foxl1-lineage cells for chemokine and cytokine signaling, and crypt-associated Foxl1-lineage cells for complement cascade components that support antibody- and phagocyte-mediated clearance of pathogens and damaged cells. These patterns suggest that Foxl1-lineage cells contribute to region-specific immune functions, including protection of the crypt stem cell compartment.

Growth factor expression further emphasized functional specialization (Figure 7*C*). Crypt and villus–tip Foxl1-lineage cells were prominent sources of epidermal growth factor receptor, epiregulin, erb-b2 receptor tyrosine kinase 2, insulin-like growth factor 1, and TGFβ, factors known to regulate epithelial growth, proliferation, and differentiation.^37^, ^38^, ^39^, ^40^, ^41^, ^42^, ^43^, ^44^, ^45^, ^46^, ^47^, ^48^, ^49^, ^50^, ^51^ In contrast, villus–base and villus–mid Foxl1-lineage cells mainly expressed growth factors predicted to act on mesenchymal, endothelial, and immune cells. Villus–tip Foxl1-lineage cells were uniquely enriched for the angiogenic factors vascular endothelial growth factor (VEGF)a and VEGFb, consistent with roles in modulating villus–tip vascular fenestration,^28^ whereas VEGFc, linked to lymphagiogenesis,^52^^,^^53^ was most abundant in villus–mid Foxl1-lineage cells.

Villus–tip Foxl1-lineage cells also showed particularly high expression of transcription factors (Figure 7*D*), including Myc/Max, Maf, Mta1, Mta2, Atf1, Etv1, and Bcl6. This population expressed genes associated with pluripotency programs, cell–cycle control, and chromatin remodeling, consistent with a dynamically regulated state with potential progenitor-like features.

To facilitate translation to human studies and practical identification of Foxl1-lineage subsets, we analyzed surface-marker expression across Foxl1-lineage clusters (Figure 7*E*). Overall, these data reveal pronounced spatial zonation in Foxl1-lineage gene-expression programs along the crypt–villus axis, supporting specialized roles in ECM remodeling, epithelial morphogenesis, immune regulation, and regional growth factor signaling.

### Computational Ligand-Receptor Analysis Supports Major Signaling Pathways Between Foxl1-Lineage Cells and Epithelial Cells

**Figure 8:**
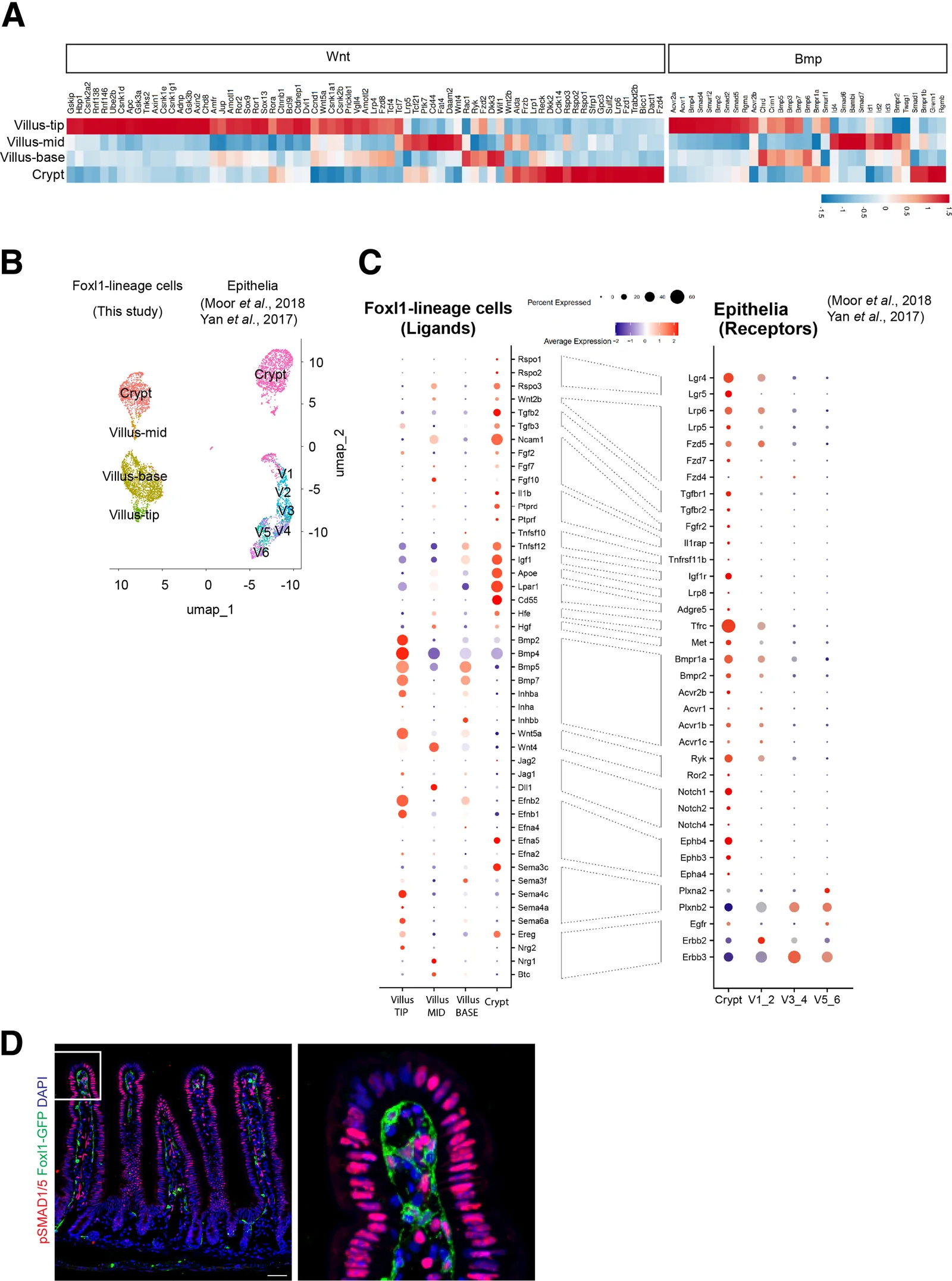
**Computational ligand-receptor analysis supports major signaling pathways between Foxl1-lineage cells and epithelial cells.** (*A*) Heatmaps of scRNA-seq data illustrate gene expression patterns in the Wnt and BMP pathways in Foxl1-lineage subtypes located at the crypt, villus–base, villus–mid, and villus–tip. (*B*) UMAP projection showing the spatial organization of Foxl1-lineage cells and epithelial cells, with each point representing a single cell color-coded by its assigned cluster. V1 to V6 correspond to epithelial regions along the villus axis from villus–base to villus–tip. (*C*) Dot plot illustrating predicted ligand–receptor interactions between Foxl1-lineage cells (ligands) and epithelia (receptors) across the crypt–villus axis. *Dashed lines* connect ligands to their corresponding receptors, indicating potential signaling interactions. (*D*) Immunofluorescence of mouse jejunum sections from Foxl1Cre; Rosa-mTmG stained for GFP and phosphorylated SMAD1/5, indicating BMP signaling activity, reveals a gradient of BMP signaling from the villus tip to crypt epithelium, with BMP activity in GFP+ villus–tip Foxl1-lineage cells. DAPI, 4′,6-diamidino-2-phenylindole.

To investigate how Foxl1-lineage cells communicate with the epithelium along the crypt–villus axis, we first examined expression of core Wnt and BMP pathway components (Figure 8*A*), which are central to intestinal zonation.^54^, ^55^, ^56^, ^57^, ^58^ Villus–tip Foxl1-lineage cells were the main source of BMP ligands. Unexpectedly, they also showed strong enrichment of Wnt pathway components, including the β-catenin-Tcf complex, Rnf146, and Tnks, consistent with potential activation of Wnt signaling via Axin-dependent mechanisms.^59^ Villus–tip Foxl1-lineage cells additionally expressed casein kinases required for canonical Wnt signaling in intestinal stem cells,^60^^,^^61^ as well as multiple subunits of the β-catenin destruction complex (Gsk3a/b, Apc, Ctnnb1, Axin1, Axin2). The upstream Wnt source for this pathway in villus–tip Foxl1-lineage cells remains unclear, but villus–mid Foxl1-lineage cells represent a plausible candidate based on their expression of canonical or dual Wnt ligands.

To systematically evaluate ligand–receptor relationships, we applied CellphoneDB^62^ ligand–receptor interaction analysis to Foxl1-lineage cells and jejunal epithelial cells^27^^,^^63^ (Figure 8*B* and *C*). Ligands enriched in crypt-associated Foxl1-lineage cells paired with receptors that were preferentially expressed in crypt epithelium, including R-spondins, Wnts, TGFβ, fibroblast growth factors, interleukin1, tumor necrosis factor, insulin-like growth factor, hepatocyte growth factor, and the iron regulator Hfe. In contrast, many ligands enriched in villus Foxl1-lineage cells had their highest matching receptor expression in crypt rather than villus epithelial cells, particularly for BMP, non-canonical Wnt, Notch, and Eph-ephrin pathways. Semaphorin and epidermal growth factor receptor signaling showed more closely matched zonated expression in both villus Foxl1-lineage cells and villus epithelium (Figure 8*C*).

To test these computational predictions, we assessed phosphorylation of SMAD1/5 as readout of BMP pathway activation. Although BMP receptors were most abundant in crypt epithelial cells and decreased toward the villus epithelium, phospho-SMAD1/5 signal was highest at the villus tip, indicating that pathway activation is primarily driven by ligand availability rather than receptor abundance. Notably, villus–tip Foxl1-lineage cells themselves displayed a graded pattern of SMAD1/5 phosphorylation (Figure 8*D*), suggesting spatially organized BMP signaling within the Foxl1-lineage network as well as in the overlying epithelium.

In summary, single-cell transcriptomics combined with spatial mapping and reporter analysis delineates 4 Foxl1-lineage populations along the crypt–villus axis, each with distinct transcriptional, matrix, immune, and growth factor signatures. Crypt-associated Foxl1-lineage cells are enriched for canonical Wnt, BMP antagonists, and niche-associated ECM programs, and show strongest predicted signaling to the crypt epithelium. Villus–base and villus–tip Foxl1-lineage cells instead exhibit higher PDGFRα, contractility-related and BMP programs, and region-specific growth factor and angiogenic profiles, whereas villus–mid Foxl1-lineage cells display immune-modulatory and inflammatory signatures. Computational ligand–receptor analyses, together with phosphor-SMAD1/5 staining, support a model in which Foxl1-lineage cells form spatially organized signaling domains that may coordinate BMP, Wnt, and other pathways between mesenchyme and epithelium along the crypt–villus axis.

## Discussion

A continuous subepithelial fibroblast network, referred as the “pericryptal mesenchymal syncytium,” has been recognized since the 1980s, based on detailed electron microscopy studies that revealed its 3-dimensional architecture.^18^^,^^64^, ^65^, ^66^, ^67^, ^68^ Despite these foundational observations, the specific cell types within this network, and their roles in regulating stem cell activity and intestinal regeneration, have remained poorly defined.

This study delineates the molecular signatures of subepithelial Foxl1-lineage populations, previously identified as telocytes, along the crypt–villus axis and suggests that they act as important coordinators of cellular growth and differentiation in the intestine. Telocytes are distinguished by their unique structural features, especially long, thin extensions (telopodes) that form an interconnected 3-dimensional meshwork beneath the epithelium.^2^^,^^4^^,^^69^, ^70^, ^71^, ^72^, ^73^, ^74^, ^75^, ^76^, ^77^ This network is not homogeneous: Foxl1-lineage telocyte populations show marked transcriptional heterogeneity, resolving into 4 spatially distinct subpopulations that group into 2 molecularly related pairs. Crypt and villus–mid Foxl1-lineage cells are enriched for canonical Wnt signaling components, whereas villus–base and villus–tip Foxl1-lineage cells predominantly express noncanonical Wnt and BMP ligands. This alternating arrangement suggests that neighboring Foxl1-lineage subtypes are interconnected and may help establish and maintain signaling gradients along the crypt–villus axis, thereby influencing epithelial cell–fate decisions and contributing to intestinal homeostasis.

The close transcriptomic similarity between villus–mid and crypt Foxl1-lineage cells, particularly their shared enrichment for canonical Wnt components, likely reflects parallel roles in supporting proliferative and protective functions within their respective epithelial territories. Although the crypt is the principal proliferative zone, villus–mid Foxl1-lineage cells may maintain a Wnt-responsive state that positions them to support rapid epithelial regeneration following injury. Because Wnt signaling intersects with major inflammatory and regenerative pathways,^78^, ^79^, ^80^, ^81^ villus–mid Foxl1-lineage cells may function as a reserve niche capable of engaging regenerative programs when tissue damage requires enhanced repair.

Our ligand–receptor analyses revealed a notable spatial pattern: ligands enriched in villus Foxl1-lineage cells often had their corresponding receptors more highly expressed in crypt epithelium than in villus epithelium. This apparent spatial separation does not necessarily imply direct villus-to-crypt signaling; rather, it highlights that receptor expression alone does not determine where pathway activity is realized. Instead, effective signaling depends on the combined distribution of ligands, inhibitors, and intersecting pathways.

BMP signaling illustrates this principle. BMP receptors are most abundant in crypt epithelial cells, yet phosphorylated SMAD1/5, the active readout of BMP signaling, shows a steep gradient with highest levels at the villus tip (Figure 8*D*). This divergence between receptor abundance and pathway activation is consistent with the zonated distribution of ligands and inhibitors: BMP ligands are enriched in villus–tip Foxl1-lineage cells, whereas BMP antagonists (Grem1, Chrd, Bambi) are concentrated in crypt, villus–base, and villus–mid Foxl1-lineage cells. Inputs from additional pathways, such as retinoic acid signaling,^82^, ^83^, ^84^, ^85^ likely further shape this landscape. Together, these features suggest that Foxl1-lineage–derived signals help establish functional BMP gradients that cannot be inferred from receptor expression alone.

Previous scRNA-seq studies of intestinal stroma typically described telocytes as a relatively uniform Foxl1+ population. However, Foxl1 is expressed at low levels and is often recorded as “zero” in droplet-based scRNA-seq, which captures only a fraction of cellular transcripts. Combined with transcriptomic similarity between telocytes and other stromal cells, this limitation has made telocytes difficult to identify without structural context. By integrating scRNA-seq with Foxl1Cre-based reporter lines and smFISH, we define 4 subepithelial Foxl1-lineage telocyte subtypes with distinct, spatially restricted expression of signaling molecules.

Our data further indicate that crypt-associated Foxl1-lineage cells, although structurally distinct, are molecularly close to neighboring mesenchymal populations such as Ackr4+ trophocytes, suggesting potential developmental or functional relationships between these stromal cell types. One possibility is that telocytes arise from other mesenchymal cells in response to local cues, acquiring characteristic morphology and signaling programs to fulfill specialized roles in tissue maintenance and regeneration.

Future work dissecting how telocytes operate as an integrated network, and how individual telocyte subtypes contribute to stem cell regulation, injury responses, and disease states, will be essential for uncovering additional mechanisms of intestinal homeostasis and regeneration.

## Materials and Methods

### Human Samples

The study on human subjects was approved by the Helsinki Institutional Review Board of the Hadassah Medical Center (HMO-0165-19). All patients signed an informed consent.

### Mice

Foxl1-Cre mice^16^ or Foxl1CreERT2 mice^19^ were crossed with the following lines:

Rosa-membrane-targeted dimer tomato protein (mT) or membrane targeted green fluorescent protein (mG) (Rosa-mTmG)^17^ (Jackson Laboratories; #007676) or Rosa-Rainbow.^22^ This produced the Foxl1Cre; Rosa-mTmG, Foxl1CreERT2; Rosa-mTmG and Foxl1CreERT2; Rosa-Rainbow.

The Ackr4-CreERT2 mouse model was generated by the Shanghai Model Organisms Center Inc using CRISPR/Cas9-mediated homologous recombination to insert a CreERT2-WPRE-polyA cassette immediately upstream of the ATG start codon of the Ackr4 gene. Ackr4-CreERT2 mice were then crossed with the Rosa-ZsGreen reporter mice (Jackson Laboratories; ME #007906) to produce Ackr4-CreERT2; Rosa-ZsGreen mice.

All animal experiments were approved by the Animal Care and Use Committee of the Hebrew University of Jerusalem.

### Tamoxifen Treatment

To induce Foxl1-CreERT2; Rosa-mTmG, Foxl1-CreERT2; Rosa-Rainbow or Ackr4-CreERT2; Rosa-ZsGreen mice, tamoxifen (Sigma-Aldrich; #10540-29-1) was dissolved in corn oil at a concentration of 30 mg/mL by shaking overnight at 37 °C. The solution was then administered intraperitoneally at a dose of 150 mg/kg body weight. Mice were injected once daily for the designated consecutive days and sacrificed for analysis.

### Tissue Fixation by Transcardiac Perfusion

Mice were anesthetized by intraperitoneal injection of ketamine/xylazine mixture (7.5% ketamine, 42.5% xylazine, v/v in saline). The thoracic cavity was opened to expose the heart, and a 23 G needle connected to a peristaltic pump was inserted into the left ventricle, followed by an incision of the right atrium to allow outflow. Phosphate-buffered saline (PBS; 10–20 mL) was first perfused to remove blood, after which 4% paraformaldehyde (PFA) in PBS was perfused at a constant flow rate. Each mouse received approximately 30 mL of fixative. Following perfusion, the jejunum or colon was dissected and post-fixed in 4% PFA at 4 °C overnight.

### Histology

Tissues were embedded in optimum cutting temperature compound (Tissue-Tek; #4586) for cryosectioning. For the Ackr4CreERT2; Rosa-ZsGreen mouse model, jejunum tissue was embedded in paraffin, and antigen retrieval was performed prior to immunostaining.

### Clearing of Human and Mouse Intestines

Intestinal tissues from both humans and mice were fixed in 4% PFA overnight at 4 °C and subsequently washed in PBS overnight at 4 °C. Tissues were then cleared for 6 hours using X-Clarity Electrophoretic Tissue Clearing (ETC) system (LOGOS Biosystems), following the manufacturer’s protocol, as previously described.^7^

### Immunofluorescence of Cleared Whole Tissue

Prior to staining, cleared intestine was blocked with 10% fetal bovine serum in PBS for 1 hour. Primary and secondary antibodies were diluted in a blocking solution containing 0.3% Triton X-100 and incubated for 48 hours at 4 °C each. Finally, the tissue was mounted en bloc on an image slide using 0.5-mm thick adhesive silicon isolator mounted in Histodenz solution (88% Histodenz [w/v] in 0.02 M PBS).

### Immunofluorescence Staining

Tissues were embedded in optimum cutting temperature compound, and antibodies were diluted in CAS-Block (Invitrogen; 008,120). Antigen retrieval for Foxl1 and C3 was performed in citrate buffer (pH 6.0; Vector; H3300) using a pressure cooker (Electron Microscopy Science). All primary and secondary antibodies were diluted in CAS-Block (Invitrogen; 008,120). For 100-μm thick sections, tissues were incubated with primary antibodies for 48 hours at 4 °C in a staining dish. Foxl1 immunostaining was performed as previously described.^9^ Antibody information is listed in Table 1.

**Table 1:** Antibody Information

Antibody	Catalogue#	Company	Dilution
Alcam	AF656-SP	Novus Biological	1:300
C3	21337-1-AP	Thermo Fisher	1:50
CD302	LS-C801732	LS Bio	1:100
CD31	557355	BD Pharmingen	1:100
CD34	14-0341-82	Thermo Fisher	1:100
CD45-APC	103112	ABCAM	1:100
CD81-APC	104909	Bio Legend	1:50
Dclk1	62257	Cell Signaling	1:300
E-Cad	610182	DB Biosciences	1:300
EpCAM	ab71916	ABCAM	1:500
Foxl1	Kind gift from Prof. Klaus Kaestner	References^7^^,^^9^	1:1500
GFP	ab6673	ABCAM	1:500
GFP	NB100-1614	Novus Biological	1:250
H3K9me3	Ab8898	ABCAM	1:100
Lyve1	MAB2125	R&D Systems	1:100
pSMAD1/5	MAB9516	Cell Signaling	1:500
mPDGFRα	AF1062	R&D Systems	1:100
hPDGFRα	AF-307-NA	R&D Systems	1:200
Postn	AF2955	R&D Systems	1:400
PDPN-APC	127418	Bio Legend	1:100
RFP	600-401-379	Thermo Fisher	1:500
αSMA	ab5694	ABCAM	1:250
Spon1 (F-Spon) R4	Kind gift from Prof. Avihu Klar	References^86^^,^^87^	1:1000
TNC	AB19011	Merck	1:300
Tuj1	BLG-801201	Bio Legend	1:500
H3K9me3	ab8898	ABCAM	1:100

### Quantification of Epithelial Cells Contacted by a Single Foxl1-Lineage Cell

To quantify the number of epithelial cells contacted by a single Foxl1-lineage cell, we analyzed jejunal sections from both Foxl1CreERT2; Rosa-mTmG mice induced with a single tamoxifen injection and Foxl1CreERT2; Rosa-Rainbow mice induced with 3 tamoxifen injections. For each reporter line, an individual Foxl1-lineage cell was identified by continuous reporter signal. For crypt region analyses, sections including the basal crypt surface were selected. The intestinal epithelium was labeled by EpCAM immunostaining. Epithelial cells were considered to be in contact with a Foxl1-lineage cell if any portion of a reporter-positive cell process made contact with an EpCAM+ epithelial cell. The total number of contacted epithelial cells for each Foxl1-lineage cell was then counted.

### Single-Molecule RNA Fluorescence In Situ Hybridization

We employed Foxl1Cre; Rosa-mTmG mice 6 to 8 weeks of age. Tissue fixation was performed in 4% PFA. Custom probe libraries, designed using the Stellaris FISH probe designer software, were utilized to hybridize with a desired coding RNA (Bio Search Technologies, Inc) and coupled to Cy5, TMR, or Alexa 594.

Cryosections (7-μm thickness) were collected on poly-L-lysine-coated slides (Sigma; #P8920) and prepared for hybridization according to the protocol^88^ with some adaptations. Briefly, tissue sections were digested with proteinase K (10 μg/mL; Ambion; AM2546) for 10 minutes and washed twice in 2× saline-sodium citrate (SSC; Ambion; AM9765). Sections were then washed in wash buffer (20% formamide [Ambion; AM9342], 2× SSC) for 5 minutes before hybridization with smFISH probes diluted 1:3000 in hybridization buffer (10% dextran sulfate [Sigma; D8906], 20% formamide, 1 mg/ml *E coli* transfer RNA (Sigma; R1753), 2× SSC, 0.02% bovine serum albumin (Ambion; AM2616), 2 mM vanadyl-ribonucleoside complex (NEB; S1402S) at 30 °C overnight. Following hybridization, sections were washed with wash buffer containing 100 ng/mL 4′,6-diamidino-2-phenylindole (Sigma-Aldrich; D9542) at 30 °C for 30 minutes. GFP antibody was diluted in the hybridization buffer, and Alexa 488 secondary antibody was diluted in the GLOX buffer (10 mM TRIS pH8.0 [Ambion; #M9856], 2× SSC, 0.4% glucose [Sigma; #G8270]) for 20 minutes at room temperature. Imaging was performed on a Nikon-A1R HD25 microscope with a 100× oil-immersion objective.

### Single-Molecule RNA Fluorescence In Situ Hybridization Quantification

The crypt–villus axis of the jejunum was divided into 4 distinct regions: crypt, villus–base (including the crypt–villus junction), villus–mid, and villus–tip, which comprises the upper one-third of the villus. Cell segmentation and automatic transcript quantification were performed in the MATLAB program (MATLAB release R2020b, MathWorks) using TransQuant transcript as described previously.^89^

### Microscopy

Microscopes used in the study:

• Nikon Eclipse Ti2 Confocal System (Nikon).
• Nikon TI2-LA-FL EPI-FL module for smFISH technology (Nikon).

Image processing was performed using Fiji, Imaris v10.0, or NIS Elements software packages.

### Single-Cell Isolation of Foxl1-Lineage Cells

Jejunum tissues were dissected from 7-8-week-old male Foxl1-Cre; Rosa-mTmG mice. Mesenchymal cells were isolated as previously described.^7^ GFP+ cells were then isolated by FACS sorting. Viable cells were gated using 4′,6-diamidino-2-phenylindole exclusion, and doublets were removed based on forward and side scatter profiles. Sorting was performed using Aria III UPG sorter. Epithelial and immune cells were excluded using EpCAM-APC and CD45-APC antibodies.

### Computational Methods

#### Single-cell RNA sequencing analysis

GFP+ sorted cells were loaded onto a Chromium single-cell 3′ v2 chip (10x Genomics) or Chromium Next GEM Single Cell Gene Expression v3.1 (3′) (10x Genomics) and libraries were prepared according to the manufacturer’s protocol. The scRNA-seq libraries were sequenced on the Illumina NextSeq500 sequencing system in a 28-bp and 56-bp as paired-ended. Seven single-cell datasets (Chromium; 10x Genomics) were filtered, retaining cells with less than 5% mitochondrial gene transcripts, more than 200 but fewer than 6000 detected genes, and 60,000 unique molecular identifiers. The resulting dataset included 256, 638, 326, 711, 253, 1204, and 4222 cells per sample. Data were normalized using the Seurat SCTransform (v2) function. Integration was performed using the IntegrateData function in Suerat, generating an integrated object comprising 7610 cells and 20,292 genes. Principal component analysis (PCA) and Uniform Manifold Approximation and Projection (UMAP) were used for dimensionality reduction. Clustering identified 18 clusters at a resolution of 0.4, using the first 50 PCA dimensions and 3000 integration features. Differentially expressed genes were identified by comparing each Foxl1-lineage cluster with the other 3 clusters. The resulting gene lists were analyzed for functional enrichment analysis using the ggplot2 R package to identify unique Gene Ontology terms. The Gene Ontology annotation displayed in the figure was selected manually to ensure a diverse representation of biological processes and to minimize repetitive or redundant terms. This approach was taken to provide a clearer and more informative summary of the functional enrichment analysis results.

#### Comparing this study dataset with that of McCarthy et al

Mouse small intestine whole-mesenchyme scRNA-seq datasets (GEO samples GSM4196131 and GSM4196132) were processed following the approach of McCarthy et al. Cells were retained if they showed less than 10% mitochondrial transcript content, contained more than 1500 detected transcripts, and included genes expressed in ≥100 single cells. Sequencing data were analyzed using Seurat (version 5.2.1) in R (version 4.4.3). After individual filtering, datasets were merged, normalized, log-transformed, and scaled. Clustering was performed using the first 50 principal components at a resolution of 0.4, yielding 20 clusters. Clusters expressing the hematopoietic marker Cd45 (Ptprc) were excluded. This resulted in 14 clusters comprising 3803 cells, 6 of which were identified as Foxl1-lineage populations based on marker gene expression. These clusters were further subsetted, and module scores for this study clusters 2, 0, 15, and 11 were calculated using Seurat’s AddModuleScore function. For each Foxl1-lineage cluster, the top 50 differentially expressed genes (compared with the other 3 Foxl1-lineage clusters) were identified, and their module scores visualized using Seurat’s RidgePlot function.

#### Foxl1-lineage cells–epithelia ligand–receptor analysis

scRNA-seq datasets of Lgr5-eGFP-negative cells (Chromium, 10x Genomics; accessions GSM2644349 and GSM2644350)^63^ were merged using the Seurat “merge” function. Based on zonation profiles reconstructed as described by Moor et al,^27^ 1383 cells were assigned to either the crypt or 1 of 6 villus zones (V; from villus–base to villus–tip): crypt (235 cells), V1 (247), V2 (168), V3 (68), V4 (312), V5 (209), and V6 (144).

The Lgr5-eGFP-positive compartment of these datasets (Chromium; 10x Genomics; accessions GSM2644349 and GSM2644350) was filtered to retain only cells with less than 10% mitochondrial gene transcripts and more than 100 but fewer than 5000 detected genes. After filtering, 638 and 778 cells from each dataset were added to the 235 Lgr5-eGFP-negative cells annotated as crypt.

The Foxl1-lineage clusters (2, 0, 15, and 11) were combined with the Lgr5-eGFP-negative and the 2 Lgr5-eGFP-positive epithelial cells into a single Seurat object (totaling 5608 cells). The data were normalized using Seurat SCTransform (v2), and PCA and UMAP were performed using the first 30 principal components.

Visualization in low-dimensional space (UMAP), as well as dot plots, violin plots, and heatmaps, were performed using Seurat’s built-in visualization functions. Figures were further customized using the ggplot2 R package6 as needed. For bulk RNA-seq analysis, scaled data were retrieved from the data layer of the dot plot objects. Heatmaps of these scaled counts were created using a custom wrapper script for the pheatmap R function. Differentially expressed genes between selected clusters were identified using Seurat’s FindMarkers function, which applies the Wilcoxon rank-sum test by default. Single-cell analysis was performed using Seurat version 5.2.0^90^, ^91^, ^92^ in R version 4.4.3.

#### CellphoneDB predicted ligand–receptor interactions

Relevant cell–cell interactions were classified into T|E, involving Foxl1-lineage cells/telocytes and epithelial clusters. For each group, dot plots were generated based on either significant mean expression (mean values are shown only if *P* < .05).

### Statistics

Statistical and graphical data analyses were conducted using GraphPad Prism 9.5.1. The number of replicates for each experiment and the statistical tests performed are detailed in figure legends.

## Supplementary Material

Supplementary Table 2

Supplementary Video 1 **A single Foxl1-lineage cell contacts the crypt base epithelium.** Immunofluorescence of a 30-μm longitudinal section from the jejunum of a Foxl1CreERT2; Rosa-mTmG mouse, following a single, low-efficiency tamoxifen induction, highlights the structure and spatial distribution of individual Foxl1-lineage cells at the crypt region.

Supplementary Video 2 **Foxl1-lineage cells contact the villus epithelium.** Immunofluorescence of a 30-μm longitudinal section from the jejunum of a Foxl1CreERT2; Rosa-mTmG mouse, following a single, low-efficiency tamoxifen induction, highlights the structure and spatial distribution of Foxl1-lineage cells along the villus region.

Supplementary Video 3 **Foxl1-lineage cells interconnect along the villus epithelium.** Immunofluorescence of a 30-μm longitudinal section from the jejunum of a Foxl1CreERT2; Rosa-Rainbow mouse, following 3 tamoxifen inductions, highlights the interconnected subepithelial network of Foxl1-lineage cells along the villus region.

## References

[1] Popescu L.M., Faussone-Pellegrini M.S. (2010). TELOCYTES - a case of serendipity: the winding way from Interstitial Cells of Cajal (ICC), via Interstitial Cajal-Like Cells (ICLC) to TELOCYTES. J Cell Mol Med.

[2] Cretoiu S.M., Popescu L.M. (2014). Telocytes revisited. Biomol Concepts.

[3] Dragos C., Maria Giuliana V., Yihua B., Zvi L. (2019). Innovations in Cell Research and Therapy.

[4] Vannucchi M.-G., Traini C., Manetti M. (2013). Telocytes express PDGFRα in the human gastrointestinal tract. J Cell Mol Med.

[5] Kondo A., Kaestner K.H. (2019). Emerging diverse roles of telocytes. Development.

[6] Shoshkes-Carmel M. (2024). Telocytes in the Luminal GI Tract. Cell Mol Gastroenterol Hepatol.

[7] Shoshkes-Carmel M., Wang Y.J., Wangensteen K.J. (2018). Subepithelial telocytes are an important source of Wnts that supports intestinal crypts. Nature.

[8] Bahar Halpern K., Massalha H., Zwick R.K. (2020). Lgr5+ telocytes are a signaling source at the intestinal villus tip. Nat Commun.

[9] Aoki R., Shoshkes-Carmel M., Gao N. (2016). Foxl1-expressing mesenchymal cells constitute the intestinal stem cell niche. Cell Mol Gastroenterol Hepatol.

[10] Kolev H.M., Kaestner K.H. (2023). Mammalian intestinal development and differentiation-the state of the art. Cell Mol Gastroenterol Hepatol.

[11] McCarthy N., Manieri E., Storm E.E. (2020). Distinct mesenchymal cell populations generate the essential intestinal BMP signaling gradient. Cell Stem Cell.

[12] Kraiczy J., McCarthy N., Malagola E. (2023). Graded BMP signaling within intestinal crypt architecture directs self-organization of the Wnt-secreting stem cell niche. Cell Stem Cell.

[13] Degirmenci B., Valenta T., Dimitrieva S. (2018). GLI1-expressing mesenchymal cells form the essential Wnt-secreting niche for colon stem cells. Nature.

[14] Paerregaard S.I., Wulff L., Schussek S. (2023). The small and large intestine contain related mesenchymal subsets that derive from embryonic Gli1(+) precursors. Nat Commun.

[15] Lemmetyinen T.T., Viitala E.W., Wartiovaara L. (2024). Mesenchymal GDNF promotes intestinal enterochromaffin cell differentiation. iScience.

[16] Sackett S.D., Fulmer J.T., Friedman J.R. (2007). Foxl1-Cre BAC transgenic mice: a new tool for gene ablation in the gastrointestinal mesenchyme. Genesis.

[17] Muzumdar M.D., Tasic B., Miyamichi K. (2007). A global double-fluorescent Cre reporter mouse. Genesis.

[18] Furuya S., Furuya K. (2007). Subepithelial fibroblasts in intestinal villi: roles in intercellular communication. Int Rev Cytol.

[19] Kolev H.M., Tian Y., Kim M.S. (2021). A FoxL1-CreERT-2A-tdTomato mouse labels subepithelial telocytes. Cell Mol Gastroenterol Hepatol.

[20] Sumigray K.D., Terwilliger M., Lechler T. (2018). Morphogenesis and compartmentalization of the intestinal crypt. Dev Cell.

[21] Srinivas S., Watanabe T., Lin C.S. (2001). Cre reporter strains produced by targeted insertion of EYFP and ECFP into the ROSA26 locus. BMC Dev Biol.

[22] Red-Horse K., Ueno H., Weissman I.L. (2010). Coronary arteries form by developmental reprogramming of venous cells. Nature.

[23] Perreault N., Katz J.P., Sackett S.D. (2001). Foxl1 controls the Wnt/beta-catenin pathway by modulating the expression of proteoglycans in the gut. J Biol Chem.

[24] Katz J.P., Perreault N., Goldstein B.G. (2004). Foxl1 null mice have abnormal intestinal epithelia, postnatal growth retardation, and defective intestinal glucose uptake. Am J Physiol Gastrointest Liver Physiol.

[25] Perreault N., Sackett S.D., Katz J.P. (2005). Foxl1 is a mesenchymal modifier of min in carcinogenesis of stomach and colon. Genes Dev.

[26] Takano-Maruyama M., Hase K., Fukamachi H. (2006). Foxl1-deficient mice exhibit aberrant epithelial cell positioning resulting from dysregulated EphB/EphrinB expression in the small intestine. Am J Physiol Gastrointest Liver Physiol.

[27] Moor A.E., Harnik Y., Ben-Moshe S. (2018). Spatial reconstruction of single enterocytes uncovers broad zonation along the intestinal villus axis. Cell.

[28] Bernier-Latmani J., Mauri C., Marcone R. (2022). ADAMTS18(+) villus tip telocytes maintain a polarized VEGFA signaling domain and fenestrations in nutrient-absorbing intestinal blood vessels. Nat Commun.

[29] Luan Y., Tang N., Yang J. (2022). Deficiency of ribosomal proteins reshapes the transcriptional and translational landscape in human cells. Nucleic Acids Res.

[30] Stzepourginski I., Nigro G., Jacob J.M. (2017). CD34+ mesenchymal cells are a major component of the intestinal stem cells niche at homeostasis and after injury. Proc Natl Acad Sci U S A.

[31] Deng M., Guerrero-Juarez C.F., Sheng X. (2022). Lepr(+) mesenchymal cells sense diet to modulate intestinal stem/progenitor cells via Leptin-Igf1 axis. Cell Res.

[32] Greicius G., Kabiri Z., Sigmundsson K. (2018). PDGFRalpha(+) pericryptal stromal cells are the critical source of Wnts and RSPO3 for murine intestinal stem cells in vivo. Proc Natl Acad Sci U S A.

[33] Iqbal S., Andersson S., Nesta E. (2025). Fetal-like reversion in the regenerating intestine is regulated by mesenchymal asporin. Cell Stem Cell.

[34] Furuya S., Naruse S., Nakayama T. (1990). 125I-endothelin binds to fibroblasts beneath the epithelium of rat small intestine. J Electron Microsc (Tokyo).

[35] Kaestner K.H., Bleckmann S.C., Monaghan A.P. (1996). Clustered arrangement of winged helix genes fkh-6 and MFH-1: possible implications for mesoderm development. Development.

[36] Madison B.B., McKenna L.B., Dolson D. (2009). FoxF1 and FoxL1 link hedgehog signaling and the control of epithelial proliferation in the developing stomach and intestine. J Biol Chem.

[37] Childs C.J., Holloway E.M., Sweet C.W. (2023). EPIREGULIN creates a developmental niche for spatially organized human intestinal enteroids. JCI Insight.

[38] Childs C.J., Poling H.M., Chen K. (2025). Coordinated differentiation of human intestinal organoids with functional enteric neurons and vasculature. Cell Stem Cell.

[39] Depeille P., Roose J.P. (2015). Flavors of EGFR-Ras signals impacting intestinal homeostasis. Cell Cycle.

[40] Dube P.E., Forse C.L., Bahrami J. (2006). The essential role of insulin-like growth factor-1 in the intestinal tropic effects of glucagon-like peptide-2 in mice. Gastroenterology.

[41] Dunker N., Schmitt K., Schuster N. (2002). The role of transforming growth factor beta-2, beta-3 in mediating apoptosis in the murine intestinal mucosa. Gastroenterology.

[42] Gough N.R., Xiang X., Mishra L. (2021). TGF-beta signaling in liver, pancreas, and gastrointestinal diseases and cancer. Gastroenterology.

[43] Kuemmerle J.F. (2012). Insulin-like growth factors in the gastrointestinal tract and liver. Endocrinol Metab Clin North Am.

[44] Laforest A., Aparicio T., Zaanan A. (2014). ERBB2 gene as a potential therapeutic target in small bowel adenocarcinoma. Eur J Cancer.

[45] Lee D., Pearsall R.S., Das S. (2004). Epiregulin is not essential for development of intestinal tumors but is required for protection from intestinal damage. Mol Cell Biol.

[46] Miguel J.C., Maxwell A.A., Hsieh J.J. (2017). Epidermal growth factor suppresses intestinal epithelial cell shedding through a MAPK-dependent pathway. J Cell Sci.

[47] Van Landeghem L., Santoro M.A., Mah A.T. (2015). IGF1 stimulates crypt expansion via differential activation of 2 intestinal stem cell populations. FASEB J.

[48] Zhang C., Jin Y., Marchetti M. (2022). EGFR signaling activates intestinal stem cells by promoting mitochondrial biogenesis and beta-oxidation. Curr Biol.

[49] Zhang X., Bandyopadhyay S., Araujo L.P. (2020). Elevating EGFR-MAPK program by a nonconventional Cdc42 enhances intestinal epithelial survival and regeneration. JCI Insight.

[50] Zhang Y., Dube P.E., Washington M.K. (2012). ErbB2 and ErbB3 regulate recovery from dextran sulfate sodium-induced colitis by promoting mouse colon epithelial cell survival. Lab Invest.

[51] Zheng Y., Song Y., Han Q. (2018). Intestinal epithelial cell-specific IGF1 promotes the expansion of intestinal stem cells during epithelial regeneration and functions on the intestinal immune homeostasis. Am J Physiol Endocrinol Metab.

[52] Nurmi H., Saharinen P., Zarkada G. (2015). VEGF-C is required for intestinal lymphatic vessel maintenance and lipid absorption. EMBO Mol Med.

[53] D'Alessio S., Correale C., Tacconi C. (2014). VEGF-C-dependent stimulation of lymphatic function ameliorates experimental inflammatory bowel disease. J Clin Invest.

[54] Qi Z., Li Y., Zhao B. (2017). BMP restricts stemness of intestinal Lgr5(+) stem cells by directly suppressing their signature genes. Nat Commun.

[55] Kosinski C., Li V.S., Chan A.S. (2007). Gene expression patterns of human colon tops and basal crypts and BMP antagonists as intestinal stem cell niche factors. Proc Natl Acad Sci U S A.

[56] Haramis A.P., Begthel H., van den Born M. (2004). De novo crypt formation and juvenile polyposis on BMP inhibition in mouse intestine. Science.

[57] Bonis V., Rossell C., Gehart H. (2021). The intestinal epithelium - fluid fate and rigid structure from crypt bottom to villus tip. Front Cell Dev Biol.

[58] Beumer J., Puschhof J., Yengej F.Y. (2022). BMP gradient along the intestinal villus axis controls zonated enterocyte and goblet cell states. Cell Rep.

[59] Callow M.G., Tran H., Phu L. (2011). Ubiquitin ligase RNF146 regulates tankyrase and Axin to promote Wnt signaling. PLoS One.

[60] Morgenstern Y., Das Adhikari U., Ayyash M. (2017). Casein kinase 1-epsilon or 1-delta required for Wnt-mediated intestinal stem cell maintenance. EMBO J.

[61] Swiatek W., Tsai I.C., Klimowski L. (2004). Regulation of casein kinase I epsilon activity by Wnt signaling. J Biol Chem.

[62] Troule K., Petryszak R., Cakir B. (2025). CellPhoneDB v5: inferring cell-cell communication from single-cell multiomics data. Nat Protoc.

[63] Yan K.S., Gevaert O., Zheng G.X.Y. (2017). Intestinal enteroendocrine lineage cells possess homeostatic and injury-inducible stem cell activity. Cell Stem Cell.

[64] Desaki J., Shimizu M. (2000). A re-examination of the cellular reticulum of fibroblast-like cells in the rat small intestine by scanning electron microscopy. J Electron Microsc (Tokyo).

[65] Desaki J., Fujiwara T., Komuro T. (1984). A cellular reticulum of fibroblast-like cells in the rat intestine: scanning and transmission electron microscopy. Arch Histol Jpn.

[66] Deane H.W. (1964). Some electron microscopic observations on the lamina propria of the gut, with comments on the close association of macrophages, plasma cells, and eosinophils. Anat Rec.

[67] Takahashi-Iwanaga H., Fujita T. (1985). Lamina propria of intestinal mucosa as a typical reticular tissue. A scanning electron-microscopic study of the rat jejunum. Cell Tissue Res.

[68] Toyoda H., Ina K., Kitamura H. (1997). Organization of the lamina propria mucosae of rat intestinal mucosa, with special reference to the subepithelial connective tissue. Acta Anat (Basel).

[69] Faussone-Pellegrini M.S., Gherghiceanu M. (2016). Telocyte's contacts. Semin Cell Dev Biol.

[70] Pieri L., Vannucchi M.G., Faussone-Pellegrini M.S. (2008). Histochemical and ultrastructural characteristics of an interstitial cell type different from ICC and resident in the muscle coat of human gut. J Cell Mol Med.

[71] Vannucchi M.-G., Traini C., Guasti D. (2014). Telocytes subtypes in human urinary bladder. J Cell Mol Med.

[72] Creţoiu S.M., Creţoiu D., Popescu L.M. (2012). Human myometrium – the ultrastructural 3D network of telocytes. J Cell Mol Med.

[73] Popescu L.M., Manole C.G., Gherghiceanu M. (2010). Telocytes in human epicardium. J Cell Mol Med.

[74] Hinescu M.E., Gherghiceanu M., Suciu L. (2011). Telocytes in pleura: two- and three-dimensional imaging by transmission electron microscopy. Cell Tissue Res.

[75] POPESCU L.M., CIONTEA S.M., CRETOIU D. (2007). Interstitial Cajal-like cells in human uterus and fallopian tube. Ann N Y Acad Sci.

[76] Hinescu M.E., Popescu L.M., Gherghiceanu M. (2008). Interstitial Cajal-like cells in rat mesentery: an ultrastructural and immunohistochemical approach. J Cell Mol Med.

[77] Rusu M.C., Loreto C., Mănoiu V.S. (2014). Network of telocytes in the temporomandibular joint disc of rats. Acta Histochem.

[78] Moparthi L., Koch S. (2019). Wnt signaling in intestinal inflammation. Differentiation.

[79] You J., Nguyen A.V., Albers C.G. (2008). Wnt pathway-related gene expression in inflammatory bowel disease. Dig Dis Sci.

[80] Hao J., Liu C., Gu Z. (2024). Dysregulation of Wnt/beta-catenin signaling contributes to intestinal inflammation through regulation of group 3 innate lymphoid cells. Nat Commun.

[81] de Vries M.H., Kuijk E.W., Nieuwenhuis E.E.S. (2025). Innate immunity of the gut epithelium: Blowing in the WNT?. Mucosal Immunol.

[82] Sheng N., Xie Z., Wang C. (2010). Retinoic acid regulates bone morphogenic protein signal duration by promoting the degradation of phosphorylated Smad1. Proc Natl Acad Sci U S A.

[83] Naylor R.W., Skvarca L.B., Thisse C. (2016). BMP and retinoic acid regulate anterior-posterior patterning of the non-axial mesoderm across the dorsal-ventral axis. Nat Commun.

[84] Miyauchi H., Ohta H., Nagaoka S. (2017). Bone morphogenetic protein and retinoic acid synergistically specify female germ-cell fate in mice. EMBO J.

[85] Bernheim S., Meilhac S.M. (2020). Mesoderm patterning by a dynamic gradient of retinoic acid signalling. Philos Trans R Soc Lond B Biol Sci.

[86] Burstyn-Cohen T., Tzarfaty V., Frumkin A. (1999). F-Spondin is required for accurate pathfinding of commissural axons at the floor plate. Neuron.

[87] Zisman S., Marom K., Avraham O. (2007). Proteolysis and membrane capture of F-spondin generates combinatorial guidance cues from a single molecule. J Cell Biol.

[88] Farack L., Itzkovitz S. (2020). Protocol for Single-Molecule Fluorescence In Situ Hybridization for Intact Pancreatic Tissue. STAR Protoc.

[89] Bahar Halpern K., Itzkovitz S. (2016). Single molecule approaches for quantifying transcription and degradation rates in intact mammalian tissues. Methods.

[90] Butler A., Hoffman P., Smibert P. (2018). Integrating single-cell transcriptomic data across different conditions, technologies, and species. Nat Biotechnol.

[91] Satija R., Farrell J.A., Gennert D. (2015). Spatial reconstruction of single-cell gene expression data. Nat Biotechnol.

[92] Stuart T., Butler A., Hoffman P. (2019). Comprehensive Integration of Single-Cell Data. Cell.

